# Translation and cross-cultural adaptation of the therapeutic intervention manual Rapid Syllable Transition Treatment (ReST) into brazilian portuguese

**DOI:** 10.1590/2317-1782/20212021257en

**Published:** 2023-03-17

**Authors:** Beatriz de Oliveira, Aline Mara de Oliveira

**Affiliations:** 1 Departamento de Fonoaudiologia, Centro de Ciências da Saúde - CCS, Universidade Federal de Santa Catarina - UFSC - Florianópolis (SC), Brasil.

**Keywords:** Apraxia, Language Therapy, Children's Language, Speech Therapy, Speech Disorders

## Abstract

**Purpose:**

To translate and culturally adapt the intervention manual *“Rapid Syllable Transition Treatment (ReST)”* into Brazilian Portuguese (PB).

**Methods:**

The translation and linguistic adaptation process followed the criteria proposed by Beaton and Guillemin: Stage 1 - Translation; Stage 2 - Synthesis of translations; Stage 3 - Back translation; Stage 4 - Expert committee review; Stage 5 - Pilot study; and Stage 6 - Evaluation of records by the researcher and expert committee.

**Results:**

In the adaptation stage, the need to modify terms and instructions translated into Portuguese by the translators for the clinical speech therapy context was shown for terms such as “beats,” “sounds,” and “smooth,” as well as the adequacy of the choice of vowels, since in PB there is no schwa vowel [ə], and there is no possible “equivalence” for Portuguese. The pilot study also indicated the need for a modification in the manual (Stage 5), being necessary to add one more lexical stress pattern, since in PB there are three lexical stress patterns, and not two as foreseen in the original English manual. Currently in the literature there are few intervention methods adapted to PB for children with speech motor disorders. Thus, the translated and adapted manual was based on the Italian adaptation of ReST, with three patterns of lexical stress and modifications in the selection of vowels, thus corroborating the linguistic context of Brazil.

**Conclusion:**

The *Rapid Syllable Transition Treatment (ReST)* manual is adapted to the Brazilian Portuguese linguistic context, with the intervention in the clinical setting being in person or through telecare. Hence, further research could prove its effectiveness through a larger sample of children.

## INTRODUCTION

Speech disorders result from different etiologies and affect different levels of speech production: Phonological and/or motor level. Thus, alterations are found in the phonological level, present in the phonological disorder, and/or the motor level, responsible for the execution and motor planning of speech, observed in childhood apraxia of speech and in speech motor delay. Both alterations in speech production are included in a broader list called speech sound disorders (SSD)^(1-3)^.

According to Shriberg et al.^(4,5)^, the Speech Disorders Classification System is categorized into three main types of speech disorders, namely speech delay (phonological disorder), residual speech errors, and motor speech disorders, in which the latter is divided into four types: Childhood apraxia of speech (CAS), childhood dysarthria (CD), speech motor delay (SMD), and associated CAS and CD.

Childhood apraxia of speech (CAS) is defined as a disorder in which the precision and consistency of movements underlying speech are impaired, without the presence of neuromuscular deficits. The damage occurs in the planning and/or programming of spatio-temporal parameters regarding the position of articulatory organs and the sequences of muscle movements, resulting in alterations in the production of speech sounds and in prosody. Finding preserved the motor and sensory systems, as well as the comprehension and attention skills^(1,2)^. The child with childhood apraxia of speech, when communicating, knows which words they want to emit, but are not capable of programing the articulatory structures in a sequence suitable for the production of sounds. CAS is characterized by three differential clinical findings: Alteration in coarticulation (transition of segments and syllables), variability of errors, with inconsistent exchanges or substitutions, and alteration in prosody (lexical stress)^(6)^.

Childhood dysarthria is a disorder in the mechanisms of speech execution, of neurological origin, characterized by slowness, weakness, imprecision, and incoordination of the speech muscles. Its characteristics are consistent and are altered in terms of voice, articulation, resonance, breathing, and musculature. Childhood dysarthria may also be associated with childhood apraxia of speech^(7)^.

Speech motor delay results from disorders in neuromotor execution, that is, there is a delay in the maturation of the speech motor system, causing changes in articulatory precision, speech stability, voice and prosody. The lack of dissociation of speech movements and excessive range of movements not expected for the age can also be found. The child needs to present at least four of twelve speech motor markers described as four red flags for the speech motor delay diagnosis, described as: limited range of speech motor movements; inconsistent production; production of few vowels and/or vowel distortions; production of few consonants and/or has consonant distortions; limited syllable and word structures; age-inappropriate phonological processes; atypical intonation; inadequate tone, frequency, volume, and nasality; difficulty in maintaining sound and syllable integrity with increased utterance length and complexity; increased variability of errors; articulatory groping; fatigue and speech unintelligibility^(5,8,9)^.

The speech therapy interventions aimed at this group of children found in the literature are associated with intensive treatment, which has been recommended for the treatment of speech sounds, specifically speech motor disorders^(10)^. Intensive therapy consists in the application of Motor Learning principles, described as: Type of feedback - feedback of Performance information (*”you didn't show that open smile”*) or Outcome information (IR, *“correct,” “almost correct”*); *feedback* frequency - high frequency during pre-practice phase and gradual decrease during practice phase; practice variability - varying parameters such as rhythm, speech rate, intensity, timbre, and vocal tone; and the increase in the complexity of the stimulus based on the child's performance^(11)^.

The scarcity of speech therapy treatment methodologies aimed at the treatment of children with speech motor disorders justifies the elaboration and/or adaptation of intervention models that are anchored in the linguistic criteria of Brazilian Portuguese, which is expected to be more effective in the treatment.

The *Rapid Syllable Transition Training* (ReST)^(12-15)^ was designed to directly address the underlying speech planning and motor programming problems experienced by Australian children with Childhood Apraxia of Speech, particularly with lexical stress management and transition from the sequence of segments or from syllable to syllable. With the aim of working on the principles of motor learning (neuroplasticity), so that each treatment session was designed to have two parts: First, a Training phase (called pre-practice in the motor learning literature), and second, a Practice phase (also called practice in the motor learning literature).

The treatment with ReST^(12-15)^ consists of high-intensity practice of twenty randomly presented pseudowords, with variations in phonetic structure and lexical stress. The twenty pseudowords are presented in four to five blocks in the practice phase, totaling one hundred productions in each session. The use of pseudowords allows children to practice motor planning and programming on word-like forms, without interference from previously incorrectly learned plans. ReST is designed to activate and use components of the speech motor system without reference to the broader language system (except the Phonology subsystem), through pseudowords listed for each child. The goal is to create new motor plans, practice them frequently, and use *feedforward* (orientation for future productions) and *feedback* (immediate response to the child's production performance) to improve the pseudowords production, aiming to improve the child's ability to use the effective speech planning and programming.

Currently, the ReST intervention method is available for Australian English, which has been originally developed to meet the demand of Australian children with CAS, and, more recently, has been adapted to Italian^(16)^.

However, to encourage the treatment dissemination, the authors^(12-15)^ explain on their website the resources development freedom for ReST in other languages by other speech-language pathologists in other countries.

Therapy with ReST has also been effective in the telecare modality. A study was carried out with five children, aged 5 to 11 years old, diagnosed with CAS, who underwent ReST therapeutic intervention through videoconference for twelve sessions (four times a week for three weeks). The results showed significant acquisition of imitation of selected pseudowords and generalization of the treatment effect to untreated pseudowords and real words. These results suggest that telecare with ReST therapy as an intervention method can be beneficial for children with CAS^(17)^.

Thus, the purpose of this study was to translate and culturally adapt the *Rapid Syllable Transition Treatment (ReST)* intervention manual into Brazilian Portuguese (PB)^(15)^.

## METHODS

This research was approved by the Research Ethics Committee of the Universidade Federal de Santa Catarina (CAAE: 35360620.9.0000.0121 and opinion no. 4.279.198/2020). The guardians of the children included in this study signed a Free and Informed Consent Form (FIC). The original version of the *Rapid Syllable Transition Treatment (ReST)* intervention method manual has been translated into Brazilian Portuguese, with the consensus and approval of the method's original authors^(12-15)^.

### Translation and cultural adaptation

To carry out the cultural adaptation of the ReST intervention method manual, the criteria proposed by Beaton et al.^(18)^ was adopted: Stage 1 - Translation; Stage 2 - Synthesis; Stage 3 - Back translation; Stage 4 - Expert Committee Review; Stage 5 - Pilot study; and Stage 6 - Evaluation and appreciation of written reports. The description of each stage was based on Beaton et al.^(18)^ and Guillemin et al.^(19)^.

1. Translation (stage 1)

The original manual was translated into the target language, Brazilian Portuguese, by three translators (Translator 1 - T1; Translator 2 - T2, and Translator 3 - T3). Beaton et al.^(18)^ recommend that at least two translations are carried out and that each translator details, in writing, the translation stages, the doubts and difficulties faced. At least one translator must know the purpose of the procedure and be familiar with academic language, while the others must not have knowledge of the procedure purpose or know the topic. This second professional is called, by Beaton et al.^(18)^, a “naive translator,” because, as they are not influenced by the academic environment, they will offer a translation capable of reflecting the language used by the target population, often highlighting ambiguous meanings of the original procedure.

Therefore, this step was carried out by three native Brazilian translators, two of whom live in the northeast region of Brazil and one in the south region of Brazil, with mastery of both languages, English and Portuguese. Two translators are bilingual undergraduates of Portuguese-English language degree, who already work in the field of translation, while the other translator is a Speech-Language Pathologist specializing in the field of language, especially in the field of speech motor disorders. The purpose of the procedure was only explained to the last translator.

The translators were asked to carry out the instrument's Portuguese version independently, preserving the semantic equivalence of all items in the original procedure.

1. Synthesis of translations (stage 2)

According to Beaton et al.^(18)^ this stage refers to the synthesis of the translations in a single version, requiring the translators to reach a consensus on their differences. To obtain the consensus version, the three translators met remotely with the researchers who followed this version development process, helping the translators with any doubts. The linguistic variations of PB were considered (linguistic adaptations, see Chart 1). The phonological variation is not an impediment to the linguistic adaptation of the instrument, since it is believed that it is not possible to contemplate all the varieties of PB present in Brazil, with adaptations by the clinician being necessary for adaptations of segments that they deem necessary in the composition of pseudowords. At the end of this stage, the first version of the procedure translation was obtained.

**Chart 1:** Comparison of the original manual, literal translation, and cultural adaptation made to the terms and instructions of the original manual in order to adapt to the linguistic and cultural reality of Brazilian Portuguese

**Original manual**	**Literal translation**	**Cultural adaptation**
Sounds	Sons	Phonemes
Beats	Batidas	Lexical stress
Smooth	Suavidade	Coarticulation
1. Choose **three long vowels** from the child’s repertoire.	1. Escolha **três vogais longas** do repertório da criança.	**Escolha 4 vogais do repertório da criança.**
		***(Choose 4 vowels from the child's repertoire.)***
2. Also select **one weak or neutral vowel (e.g. schwa).**	2. Escolha também **uma vogal fraca ou neutra** (ex: schwa).	Keeping in mind that if the therapist chooses /e/ or /o/ in a final unstressed syllable position, in some Brazilian regions, it will be pronounced as [I] and [U], respectively
3. For some children in the research we selected **two long, one short and schwa.**	3. Para algumas crianças da pesquisa nós selecionamos **duas vogais longas, uma curta e schwa.**	It is suggested that the therapist selects the vowels /a/, /i/, /u/ due to the oppositions (extremities of the vowel triangle) of the articulatory gestures involving the three vowels.

The words that have been translated and adapted are in bold

1. Back translation (stage 3)

After completing the necessary cultural adaptations, a native translator from the United States carried out the back translation (Portuguese - English). The translator was not the same from the first stage and had no knowledge of the purposes of the procedure; therefore, she worked on the back translation into English without having access to the original version.

The purpose of this stage was to verify if that version would reflect the content from the original version. Then, the original version and the back translation were compared and corrected so that translation and interpretation errors were excluded. And to verify that the adapted version did not change the significance of the original content.

1. Expert committee review (stage 4)

At this stage, according to Beaton et al.^(18)^, a consensus must be reached, based on all the versions of the translations produced, so that a pre-final version is prepared.

The materials made available to the expert committee were the original manual in English, the translations, and the synthesis of the translations. This stage aims to make the necessary changes in the intervention manual to ensure both cultural and semantic, idiomatic and conceptual equivalence.

The work on semantic, cultural, idiomatic, and conceptual equivalence was carried out with the help of two linguistics PhD students. At this stage, the study researchers met remotely with the expert committee to analyze the original procedure and all the versions produced in order to prepare a pre-Final version.

1. Application of the pre-final version of the pilot study manual (stage 5)

Pilot study with the aim of detecting possible errors, evaluating the quality of the adaptation and the practical aspects of its application. This stage allows polishing the manual writing (Supplementary Material), as well as its cultural adaptation and validation. The pilot study participants are two patients from the School Clinic of Speech Therapy at the Universidade Federal de Santa Catarina, who participate in the Phonological Disorder and Apraxia Research Project (CAAE: 35360620.9.0000.0121 and opinion no. 4.279.198/2020).

The following inclusion criteria were adopted: 1) having been previously attended at the Universidade Federal de Santa Catarina; 2) having a diagnosis of severe speech sound disorders; no associated comorbidities and no hearing loss; 3) having parents or guardians signing the Free and Informed Consent Form; 4) signature of the Term of Agreement. The following were excluded: 1) individuals with mild speech sound disorders; 2) patients with associated comorbidities such as intellectual disability, syndromes, or hearing loss. This information was observed in the patient's medical record or obtained from anamnesis.

Initially, the phonological assessments were carried out: Phonological Assessment based on the Phonological Assessment of Child Speech instrument^(20)^. And the specific assessments of motor speech production: Assessment of Multisyllabic Words; Phrasal Stress Assessment; Speech Inconsistency Assessment and Speech Motor Assessment^(21)^. Then, the application of the ReST Intervention Method was implemented in the two subjects through telecare.

The version of the manual adapted to Portuguese of the ReST intervention method^(12-15)^ was applied for twelve intensive telecare sessions in two siblings with speech sound disorders, respectively: S1- 9-year-old child diagnosed with speech motor delay and S2- 16-year-old adolescent diagnosed with childhood apraxia of speech. Patients were reassessed one week and one month after the end of the intervention sessions using the Child Phonological Assessment instrument and the Percentage of Consonants Correct (PCC-R)^(20)^.

Evaluation and appreciation of all reports provided by the researcher and expert committee (stage 6)

All translations and back translations, as well as the material used for the pilot study and the material with the corrections made after this stage, were sent to the audit process in order to verify that all stages of the translation and cultural adaptation process had been followed. After this stage, changing the procedure content was not allowed.

Two Speech-Language Pathology researchers were invited to participate in the audit process, together with the specialist Speech-Language Pathologists, and were instructed on the purposes of the research. They were asked to indicate whether each stage of the translation and cultural adaptation process had been followed and whether the procedure, based on the information provided and the pilot study, was adequate for the translation and cultural adaptation of the *Rapid Syllable Transition Treatment (ReST)* therapeutic intervention manual^(12-15)^ into Brazilian Portuguese.

## RESULTS

Stage 1 (translation) and stage 2 (synthesis of translations)

Three translators did the translations separately, without communicating with each other. Decisions regarding the terms used and the best structure, as well as the translators' difficulties with the Portuguese version were described in a report for the researchers of this study. In Stage 2, the translators met with the researchers remotely and performed a single version of the three translations (synthesis of the translations). The translators reached the consensus of writing the manual in Brazilian Portuguese orthographic norms; however, certain difficulties were encountered during the process.

The “naive” translators, who had no knowledge of the intervention method and the area of study, reported difficulty in the translation process due to terms belonging to the areas of Speech Therapy, Phonetics and Phonology. When translating, it was necessary to research the equivalent terms in Portuguese that were relevant to the official term.

Another difficulty found by the translators was the translation of the manual’s practice phase, in which the phonemes belonged to the English language, such as the vowel schwa [ə], not being possible to make an “equivalence” for Portuguese without the help of a speech therapist and specialist in the field during this step.

The bilingual translator, speech-language therapist reported the difficulty in some terms that were used in English which did not have corresponding words in Portuguese that represented the same speech-language pathology terms used in Brazil, such as the terms *“sounds”, “beats,”* and *“smooth”* (Chart 1).

Together with the researchers, they decided to modify some terms and instructions translated into Portuguese by the translators in the context of the language used in clinical speech therapy practice. In this way, the cultural adaptation of the manual was carried out, as shown in Chart 1.

Examples of ready-made pseudowords, phrases and lists were also necessary to be modified during the adaptation, considering that they could not be used as they were not part of the Brazilian Portuguese linguistic context; therefore, they were replaced in the manual by examples suitable for Brazilian Portuguese.

After finalizing the single version, the researchers made adjustments to the instructions, phrases and texts to facilitate the understanding of the manual. After this stage, we proceeded to stage 3 - back translation.

Stage 3 (back translation)

At this stage, after the necessary cultural adaptations, a fourth translator, a native English translator, performed the back translation (Portuguese - English), in this way it was verified that the instructions and texts of the manual translated and adapted to Brazilian Portuguese reflected the content of the original version. Only the modified steps during the adaptation, as shown above, differed from the original; however, they did not imply the full significance of the original content.

Stage 4 (expert committee review)

At this stage, the study researchers met remotely with the expert committee to review the original manual and all versions produced in order to prepare a pre-Final version. Thus, the judges agreed that the instructions and texts reflect the original version content, as well as the adaptations proposed in the previous steps proved to be adequate to the Brazilian linguistic context and meet the demand for cultural adaptation to finally be applied to children with speech sound disorders.

Stage 5 (application of the pre-final version of the manual - pilot study)

The pilot study carried out with two patients demonstrated the need to modify the manual with the addition of another lexical stress pattern in order to corroborate the frequency of lexical stress present in Brazilian Portuguese; therefore, the Italian adaptation version was consulted^(16)^ following the manual authors' guidance^(12-15)^ to carry out the adaptation to Brazilian Portuguese. Hence, the composition of the twenty pseudowords was organized according to Table 1.

**Table 1:** Cultural adaptations of lexical stress patterns

**Original manual**	**Literal translation**	**Cultural adaptation**
**Create 10 words with the format of [Sww]-** Strong syllable (long vowel), Weak syllable [schwa or short vowel], Weak syllable [schwa/short vowel]	**Crie dez pseudopalavras com o formato [Fff]** - sílaba Tônica (vogal longa), sílaba Átona (schwa/vogal curta), sílaba Átona (schwa/vogal curta)	**Crie 7 pseudopalavras no formato [fraco-Forte-fraco]** (paroxítona)
		***(Create 7 pseudowords in the format [weak-Strong-weak]*** *(paroxytone))*
		**Crie 7 pseudopalavras no formato [fraco-fraco-Forte]** (oxítona)
		***(Create 7 pseudowords in the format [weak-weak-Strong]*** *(oxytone))*
**Create 10 words with the format of [wSw]** -Weak syllable [schwa], Strong syllable (long vowel), Weak syllable [schwa/short vowel]	**Crie dez pseudopalavras com o formato [fFf]** -sílaba Átona (schwa), sílaba Tônica (vogal longa), sílaba Átona (schwa/vogal longa)	**Crie 6 pseudopalavras com o formato [Fff]** (proparóxitona)
		***(Create 6 pseudowords with the format [Fff]*** *(proparoxytone)*

The words that have been translated and adapted are in bold

**Figure 1:**
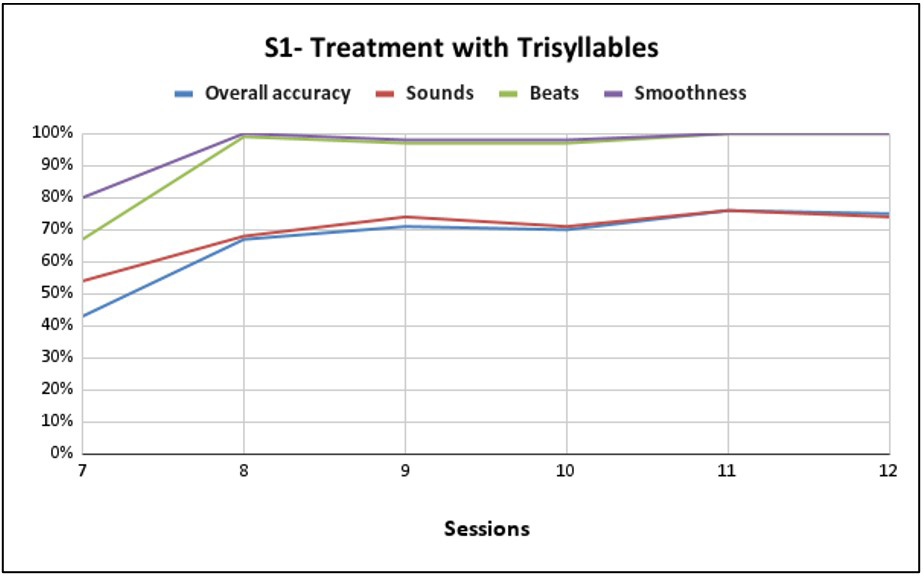
Performance of S1 disyllable training with pseudowords composed of /d/, /k/, /l/, /f/, /a/, /i/, /u/, /e/

**Figure 2:**
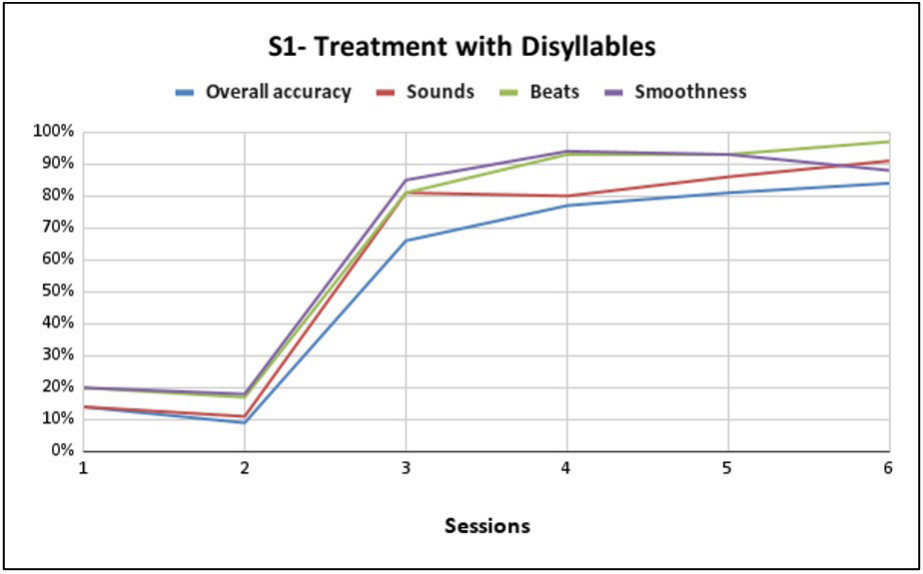
Performance of S1 trisyllabic training with pseudowords composed of /d/, /k/, /l/, /f/, /a/, /i/, /u/, /e/

**Figure 3:**
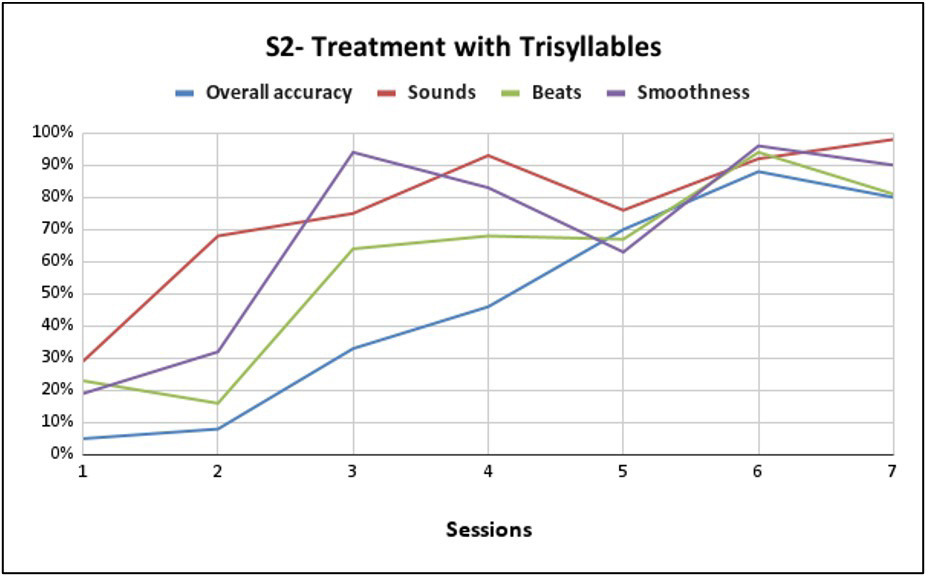
Performance of S2 trisyllabic training with pseudowords composed of /k/, /l/, /s/, /ʒ/ /a/, /i/, /u/, /e/

**Figure 4:**
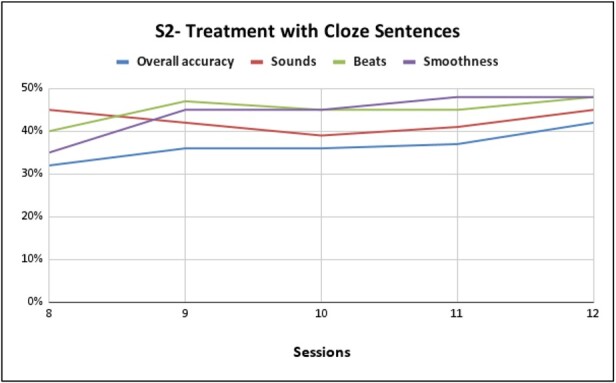
Performance of S2 Cloze sentence training with pseudowords composed of /k/, /l/, /s/, /ʒ/ /a/, /i/, /u/, /e/

In the study, the subjects showed good acceptance of the therapeutic intervention during the twelve telecare sessions and there were no complaints or reports of difficulty in understanding the instructions for the application of the manual. The descriptions of cases and the verification of the effectiveness of the method will be discussed in future publications, however, good performance was observed during the sessions (Figures 1, 2, 3, and 4). The two subjects showed progress in the level of complexity of the pseudowords, as they reached 80% of correct answers in two consecutive sessions.

Figure 1 shows the performance of S1 disyllabic training with pseudowords composed of /d/, /k/, /l/, /f/, /a/, /i/, /u/, /e/. Presenting a 70% gain in general production compared to the first session at the end of the sixth session, a 77% gain in phoneme performance, a 77% improvement in the production of lexical stress, and 68% in the production of coarticulation. From the seventh session, S1 had already achieved good performance and adherence to therapy, thus advancing to the trisyllabic level. During the intervention sessions, there was a 32% gain in general production at the end of the last session, a 20% gain in phoneme performance, a 33% improvement in the production of lexical stress, and a 20% improvement in the production of coarticulation, as shown in Figure 2.

As for the performance of S2 with pseudowords composed of /k/, /l/, /s/, /ʒ/ /a/, /i/, /u/, /e/, we can verify in Figure 3 a gain of 75% in general production compared to the first session at the end of the seventh session, 69% gain in phoneme performance, 58% improvement in the production of lexical stress, and 71% in the production of coarticulation. From the eighth session onwards, S2 had already achieved good performance and adherence to therapy, thus also advancing in level, as shown in Figure 4. In cloze sentences, we verified a 10% gain in general production compared to the eighth session at the end of the twelfth session, 8% improvement in the production of lexical stress, 13% in the production of coarticulation and persevering the same percentage in the performance of phonemes compared the eighth session to the end of the twelfth session.

In Chart 2 we can analyze a good prognosis during the pre-therapy reassessments and after one week and one month of therapeutic intervention, respectively. It is noteworthy that the cases will be discussed in more detail in future publications.

**Chart 2:** Analysis of subjects in pre-therapy and after twelve sessions of therapeutic intervention

**SUBJECTS**	**EVALUATION INSTRUMENTS**	**PRE-THERAPY**	**POST THERAPY - 1 WEEK**	**POST THERAPY - 1 MONTH**
	**PCC-R**	**46.56%**	**50.88%**	**48.23%**
		OI- ACQUIRED	OI- ACQUIRED	OI- ACQUIRED
		/p/, /b/, /t/, /d, /g/, /v/, /m/, /n/, /d Ʒ/, /tʃ/	/p/, /b/, /t/, /d, /g/, /v/, /m/, /n/, /d Ʒ/, /tʃ/	/p/, /b/, /t/, /d, /g/, /v/, /m/, /n/, /d Ʒ/, /tʃ/
		
		OI- NOT ACQUIRED	OI- PARTIALLY ACQUIRED	OI- PARTIALLY ACQUIRED
		/k/, /f/, /l/, /s/, /z/, /Ʒ/, /ʃ/, /R/	**/k/, /f/, /l/**	**/k/**
**S1**	**PHONOLOGICAL INVENTORY - (Phonological Assessment, YAVAS)**	OM- ACQUIRED	OM- ACQUIRED	OM- ACQUIRED
		/p, /b/, /t/, /d/, /g/, /v/, /m/, /n/, /dƷ/, /tʃ/, /ɲ/	/p, /b/, /t/, /d/, /g/, /v/, /m/, /n/, /dƷ/, /tʃ/, /ɲ/	/p, /b/, /t/, /d/, /g/, /v/, /m/, /n/, /dƷ/, /tʃ/, /ɲ/
		
		OM- NOT ACQUIRED	OM- PARTIALLY ACQUIRED	OM- PARTIALLY ACQUIRED
		/k/, /ɾ/, /l/, /f/, /s/, /z/, /R/, /Ʒ/, /ʃ/, /ʎ/	**/k/**	**/k/**
		CM- PURCHASED	CM- PURCHASED	CM- PURCHASED
		/m/, /n/,	/m/, /n/,	/m/, /n/,
		
		CM- NOT ACQUIRED	CM- NOT ACQUIRED	CM- NOT ACQUIRED
		/s/, /ʃ/, /Ʒ/, /R/	/s/, /ʃ/, /Ʒ/, /R/	/s/, /ʃ/, /Ʒ/, /R/
		
		CF- ACQUIRED	CF- ACQUIRED	CF- ACQUIRED
		/m/, /n/, /ʃ/, /R/	/m/, /n/, /ʃ/, /R/	/m/, /n/, /ʃ/, /R/
		
		CF- NOT ACQUIRED	CF- NOT ACQUIRED	CF- NOT ACQUIRED
		/s/, /Ʒ/	/s/, /Ʒ/	/s/, /Ʒ/
	**PCC-R**	**66.28%**	**82.37%**	**81.18%**
**S2**		OI- ACQUIRED	OI- ACQUIRED	OI- ACQUIRED
		/p/, /b/, /t/, /d, /g/, /v/, /m/, /n/, /dƷ/, /tʃ/, /l/, /R/	/p/, /b/, /t/, /d, /g/, /v/, /m/, /n/, /dƷ/, /tʃ/, /l/, /R/,	/p/, /b/, /t/, /d, /g/, /v/, /m/, /n/, /dƷ/, /tʃ/, /l/, /R/,
			**/k/, /Ʒ/, /s/, /f/**	**/k/, /Ʒ/, /s/, /f/**
		OI- PARTIALLY ACQUIRED		
		/k/	OI- NOT ACQUIRED	OI- PARTIALLY ACQUIRED
			/ʃ/, /z/	**/z/**
		OI- NOT ACQUIRED		OI- NOT ACQUIRED
		/f/, /s/, /z/, /Ʒ/, /ʃ/		/ʃ/
	**PHONOLOGICAL INVENTORY - (Phonological Assessment, YAVAS)**	OM- ACQUIRED	OM- ACQUIRED	OM- ACQUIRED
		/p/, /b/, /t/, /d, /g/, /v/, /m/, /n/, /ɲ/, /dƷ/, /tʃ/, /l/, /R/, /ɾ/, /ʎ/	/p/, /b/, /t/, /d, /g/, /v/, /m/, /n/, /ɲ/, /dƷ/, /tʃ/, /l/, /R/, /ɾ/, /ʎ/,	/p/, /b/, /t/, /d, /g/, /v/, /m/, /n/, /ɲ/, /dƷ/, /tʃ/, /l/, /R/, /ɾ/, /ʎ/,
			**/f/, /k/, /Ʒ/, /s/**	**/f/, /k/, /Ʒ/, /s/**
		OM- PARTIALLY ACQUIRED		
		/k/	OM- NOT ACQUIRED	OM- PARTIALLY ACQUIRED
			/z/, /ʃ/	**/z/**
		OM- NOT ACQUIRED		
		/f/, /s/, /z/, /Ʒ/, /ʃ/		OM- NOT ACQUIRED
				/ʃ/
		CM- PURCHASED	CM- PURCHASED	CM- PURCHASED
		/m/, /n/	/m/, /n/, **/s/, /ʃ/**	/m/, /n/, **/s/, /ʃ/**
		
		CM- NOT ACQUIRED	CM- NOT ACQUIRED	CM- NOT ACQUIRED
		/s/, /ʃ/, /R/	/R/	/R/
		
		CF- ACQUIRED	CF- ACQUIRED	CF- ACQUIRED
		/m/, /n/, /ʃ/, /R/, /s/	/m/, /n/, /ʃ/, /R/, /s/	/m/, /n/, /ʃ/, /R/, /s/

**Caption:** OI- Initial Onset; OM- Medial Onset; CM- Medial Coda; CF - Final Coda. In bold are the phonemes partially and/or totally acquired during the twelve intervention sessions by S1 and S2. The two subjects do not present consonant clusters in their phonological inventory

Stage 6 (Evaluation and appreciation of all reports provided by the researcher and expert committee):

With the completion of the translations, back translation, pilot study, and the necessary adjustments indicated in the pilot study, the guise of the final version of the manual was reached. Two researchers from Speech-Language Pathology in the field of Language participated in the audit process, together with the speech-language pathologists (Doctors and Masters) who composed the committee of experts and the researchers of this article to verify that the steps of the translation and adaptation process had been followed and fulfilled all criteria.

The audit process concluded that all steps were completed and that the procedure was ready to proceed to the population characterization phase.

## DISCUSSION

The purpose of this research was to translate and culturally adapt into Brazilian Portuguese (PB) the intervention manual *“ReST- Rapid Syllable Transition Treatment”*, aimed at the treatment of children with childhood apraxia of speech.

In the English, the lexical stress pattern in polysyllabic words is predominantly positioned on the antepenultimate syllable for nouns (Strong-weak-weak), on the antepenultimate and penultimate syllable for verbs (Strong-weak-weak or weak-Strong-weak)^(22)^. Thus, in the original manual, the therapeutic intervention method ReST^(12-15)^, in English, was implemented using only two lexical patterns in the language, totaling twenty pseudowords with two variations of lexical stress.

On the other hand, as in Brazilian Portuguese, the Italian language presents three patterns of lexical stress (weak-Strong-weak; weak-weak-Strong; Strong-weak-weak); thus, in the Italian adaptation the authors implemented the representation of the three lexical patterns in pseudowords as therapy stimuli^(16)^. Instead of increasing the number of pseudowords, the three lexical patterns were divided according to their frequency in the language. Whereas in Italian, more than 80% of words with at least three syllables are stressed on the penultimate syllable (weak-Strong-weak - paroxytones); 16% have lexical stress on the antepenultimate syllable (Strong-weak-weak-proparoxytone) and 4% have stress on the last syllable of the word (weak-weak-Strong-oxytone)^(23)^.

As instructed by the original authors^(12-15)^, the stage of cultural adaptation (Stage 5) to PB was also based on the adaptation from Italian^(16)^, thus, through the analysis of lexical stress patterns in Portuguese: words with at least three syllables with stress on the penultimate syllable are equivalent to about 62% (weak-Strong-weak - paroxytones); stress in the last syllable are present in 25% (weak-weak-Strong - oxytone) and the less common ones have lexical stress in the antepenultimate syllable (Strong-weak-weak-proparoxytone) make up 12%^(24)^. Thus, the authors, during the adaptation, chose to compose the 20 pseudowords organized into 7 pseudowords in the format [weak-Strong-weak] (paroxytone); 7 pseudowords in the format [weak-weak-Strong] (oxytone) and 6 pseudowords in the format [Strong-weak-weak] (proparoxytone).

As for the consonants that make up the pseudowords, no adaptation was necessary in relation to the original manual^(15)^, remaining the instruction for the selection of four consonants existing in the child's phonological inventory. Regarding vowels, as the *schwa* /ə/ is not used to indicate unstressed/weak syllables in PB, it was decided not to include them in the pseudowords stimulus list, unlike what was foreseen in the English language manual^(15)^. The oral vowels of PB, [a], [e], [ɛ], [i], [o], [ɔ] and [u] were inserted, and preferably, the choice of targets should be initiated by the vowels [a], [i] and [u] and, later, the medial vowels [e], [ɛ], [o] and [ɔ] must be inserted in the pseudowords.

The organization of the sessions, according to the original method in English,^(12-15)^ proposes a pre-practice phase consisting of training the pseudowords using any clue or feedback, and a practical phase with four to five blocks of twenty pseudowords each without specific correction feedback, with two-minute playful games in the intervals between blocks. In the application of stage 5, the organization of the sessions proved to be effective during the therapeutic interventions in the children of the pilot study. Therefore, the adaptation of the method to Brazilian Portuguese was based on the method of therapeutic intervention in English and Italian^(16)^, not requiring changes in relation to these aspects.

Studies^(25,26)^ indicate the effectiveness of intensive therapies in speech motor disorders, thus Murray et al.^(25)^ demonstrates the comparison between two intervention models ReST and NDP3 the Nuffield Dyspraxia Program-Third Edition (NDP3), and despite both showing strong evidence in treatment and generalization, ReST showed greater gains in children with childhood apraxia of speech.

Thus, confirming the study by Murray et al.^(25)^, the pilot study carried out with two children with speech sound disorders, one with speech motor delay and the other childhood apraxia of speech, who underwent intensive intervention of the ReST method for twelve sessions, demonstrated satisfactory treatment results.

A study involving a systematic review of specific treatments for CAS also demonstrated that ReST is clinically effective in the treatment of childhood apraxia of speech, as strong generalization effects were demonstrated in the treatment, evidencing greater continuation of treatment effects and generalization of non-words treated^(26)^. Therapy with ReST through the application of the intervention manual also proved to be effective in the telecare modality, corroborating studies by Thomas et al.^(17)^ with five children, aged 5 to 11 years old, diagnosed with CAS, who underwent a ReST therapeutic intervention through videoconference for twelve sessions (four times a week for three weeks). The results showed significant acquisition of imitation of selected pseudowords and generalization of the treatment effect to untreated pseudowords and real words.

Thus, after completing all the stages, it is stated that the translation and adaptation of the original manual in English into Brazilian Portuguese was satisfactory, and it is adapted to the clinical therapeutic context of Brazil, no longer being a language barrier. Although linguistic adaptations have been contemplated, it is up to the clinician to make phonetic adaptations according to the production of speakers in the region, when necessary. And with the potential to become a therapeutic resource with easy access to the clinical practice of Brazilian speech therapists, as well as in English and Italian.

We emphasize the importance of validation studies with a larger sample of children, with randomized design, with different clinical conditions, specifically childhood apraxia of speech and speech motor disorders, to verify the effectiveness of the method.

## CONCLUSION

From the translation stages and with the application of the pilot study, we could verify that the goal of translating and adapting an intervention program for Brazilian children with speech motor disorders was met. Through the adaptation stage, the need to modify terms and instructions translated into Portuguese by the translators for the context of clinical speech therapy practice was verified. The pilot study also indicated the need for modification in the manual (Stage 5), and it was necessary to add one more pattern of lexical stress, since in PB there are three patterns of lexical stress, and not two as foreseen in the original manual in English. As for the organization of the sessions provided for in the original manual, no modifications were necessary due to the good acceptance of the patients. Therefore, the *Rapid Syllable Transition Treatment* (ReST) manual is adapted to the linguistic context of Brazilian Portuguese, and it is up to the clinician to adapt, when necessary, considering the linguistic variation of the patient's region. Thus, further studies are needed to prove the effectiveness with a larger sample of children, both in the clinical group of speech motor disorder and in the other disorders involving sounds in speech.

## Appendix

This article comes with supplementary material.

This material is available at: https://rest.paginas.ufsc.br/
