# Supplementary material for: Translation and cross-cultural adaptation of the therapeutic intervention manual Rapid Syllable Transition Treatment (ReST) into brazilian portuguese
Source: Codas. 2023 Mar 17;35(2):e20210257. doi: 10.1590/2317-1782/20212021257en (PMC10029998; doi:10.1590/2317-1782/20212021257en)
Supplement: Supplementary file 1 [file codas-35-2-e20210257-suppl01-en.pdf]

THE UNIVERSITY OF SYDNEY

UNIVERSIDADE FEDERAL DE SANTA CATARINA

Manual Clínico do Método de Terapia Transição Rápida de Sílabas

(Rapid Syllable Transition Treatment -ReST)

Tradução e adaptação: Beatriz de Oliveira e Aline Mara de Oliveira

2021

Patricia McCabe, Elizabeth Murray, Donna Thomas and Pippa Evans

Tradução e adaptação: Beatriz de Oliveira e Aline Mara de Oliveira

Como desenvolver a terapia ReST?

Como saber por onde começar?

Em caso de dúvidas, comece com pseudopalavras de **três sílabas** (conforme o fluxograma demonstrará), mais precisamente:

- **Comece com três sílabas:** Caso a criança consiga produzir no mínimo cinco palavras[1] diferentes de três sílabas (por mais que não sejam 100% precisas), comece com pseudopalavras[2] compostas por três sílabas.
- **Comece por duas sílabas:** Para crianças que produzem menos de cinco palavras trissílabas ou menos de cinco palavras dissílabas, comece com pseudopalavras com duas sílabas.
- **Para crianças mais velhas:** Aquelas que apresentam somente problemas com a prosódia e palavras mais longas, comece com pseudopalavras com três sílabas, mas esteja preparado para avançar para as sentenças Cloze (mais informações adiante).

- **Se você está em dúvida entre começar com duas ou três sílabas, favor considerar:**
  - O temperamento da criança. Algumas crianças tentarão pronunciar as pseudopalavras sem muita preocupação, mas outras não tentarão caso acharem que tais palavras aparentam ser difíceis. Portanto, inicie com a criança em um nível que estimule o seu engajamento com o tratamento.
  - Preparando-as para o sucesso. Se está com dúvida se elas conseguem produzir palavras de duas ou três sílabas, começando com pseudopalavras com duas sílabas permitirá que elas aprendam o que é esperado para o tratamento antes de prosseguir para pseudopalavras com três sílabas. Pseudopalavras de duas sílabas tornam-se base para pseudopalavras de três sílabas, as de três sílabas para as pseudopalavras de quatro sílabas e/ou frases inteiras.
  - A precisão das palavras de três sílabas. As crianças deverão ser capazes de produzir pelo menos duas palavras com a estrutura CVCVCV (mesmo que produzam sem perfeição), antes de começarem com as pseudopalavras de três sílabas. Substituições frequentes (CV > V) e a não realização fonética de sílabas podem ser mais bem abordadas inicialmente com pseudopalavras de duas sílabas.

Objetivos da Terapia

O objetivo desta terapia é proporcionar que a produção de fala da criança para pseudopalavras seja igual ao de um adulto da sua comunidade de fala. Se, na Fase Prática, a criança conseguir 80% de acertos em mais de 100 tentativas e em mais de duas sessões consecutivas, ela segue para o próximo nível da hierarquia.

---

[1] Se refere ao repertório lexical da criança, o fonoaudiólogo realizará inicialmente a avaliação do mesmo, e assim, identificará a quantidade de léxico e sílabas que a criança é capaz de produzir.
[2] Palavras sem sentido as quais são utilizadas na terapia ReST.

Dentro de uma sessão, se a criança fizer dois blocos de prática com menos de 10% de acertos ao longo dos blocos, o terapeuta deverá realizar outro bloco de treinamento e então finalizar com o próximo bloco de prática. Caso a criança alcance menos de 10% de acertos na fase de treinamento de duas sessões consecutivas você deverá regredir um nível. Verifique o fluxograma para tomadas de decisões clínicas.

Não foram estabelecidos critérios de alta na pesquisa, pois cada criança só foi observada por 12 sessões, independentemente da severidade de seus casos [3].

Na próxima página, você encontrará um Fluxograma de Definição de Metas, seguido de alguns exemplos das metas.

Fluxograma de Definição de Metas para a Terapia ReST.

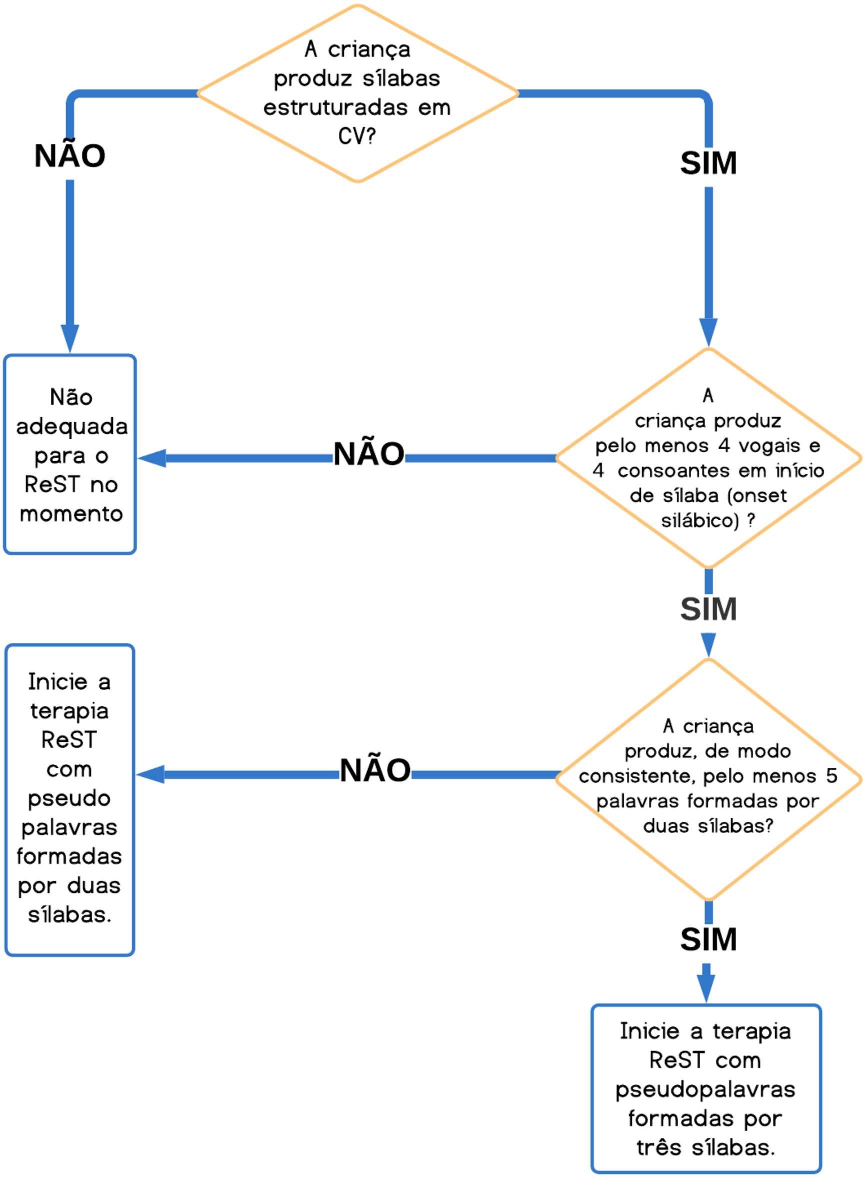

[3] Estudos da terapia Rest no inglês australiano, a pesquisa no Brasil ainda está em desenvolvimento. .

Exemplo de metas:

Meta básica: A criança produzirá pseudopalavras de três sílabas com, simultaneamente, fonemas, acento lexical e coarticulação corretas em 80% de 100 tentativas na fase prática (5 blocos x 20 pseudopalavras).

Avanço da meta básica: A criança produzirá sentenças de Cloze com pseudopalavras de três sílabas com, simultaneamente, fonemas, acento lexical e coarticulação corretas em 80% de 100 tentativas na fase de prática (5 blocos x 20 pseudopalavras).

Regressão da meta básica: A criança produzirá pseudopalavras de duas sílabas com, simultaneamente, fonemas, acento lexical e coarticulação corretas em 80% de 100 tentativas na fase prática (5 blocos x 20 pseudopalavras).

Regressão adicional: A criança produzirá pseudopalavras de duas sílabas com dois parâmetros simultaneamente corretos (exemplos: fonemas + acento lexical, fonemas + coarticulação ou acento lexical + coarticulação) em 80% de 100 tentativas na fase prática (5 blocos x 20 pseudopalavras).

Preparação dos Materiais do Tratamento

Os terapeutas podem:

1. Utilizar, sem alterações, uma lista existente de pseudopalavras (anexos 1, 2, 3 ou 4), se for adequado à criança, ou
2. Criar seus próprios materiais (veja abaixo)

Seleção de Alvos

O conjunto de pseudopalavras é composto por 4 consoantes e 4 vogais

Consoantes:

As consoantes devem estar no inventário da criança em início de sílaba (*onset* silábico).

As pseudopalavras apresentam consoantes as quais se **diferem** umas das outras – incluindo pelo menos um fone desvozeado e um fone vozeado. As pseudopalavras precisam de consoantes com pelo menos duas classes de modo de articulação – ex: plosivas, fricativas/africadas, nasais ou líquidas.

Evite sons de desenvolvimento mais tardio ou padrão silábico com estrutura CCV, que representem alguma dificuldade de produção com precisão pela criança. Portanto, se você deseja utilizar uma lista existente de pseudopalavras, substitua os sons que a criança não consegue produzir sem muita assistência por aquele que pertence à mesma classe de modo de articulação (ex: substitua /f/ por /s/, mantendo assim, a mesma classe de fricativa).

Vogais

Escolha **quatro vogais** do repertório da criança.

Sugere-se que o terapeuta selecione as vogais /a/, /i/, /u/ em decorrência das oposições (extremidades do triângulo vocálico) dos gestos articulatórios envolvendo as três vogais. Lembrando que caso o terapeuta escolha /e/ ou /o/ em posição de sílaba átona final, em algumas regiões brasileiras, será pronunciado como [I] e [U], respectivamente.

Pseudopalavras

Como descrito acima, você possui quatro opções de escolha para conjuntos de pseudopalavras para cada criança:

1. Usar um conjunto já existente sem modificação, que está disponível nos Anexos 1 e 2.
2. Usar um conjunto já existente e modificá-lo para a criança
3. Criar um conjunto novo do início
   Mais detalhes de cada opção a seguir

1. Lista de pseudopalavras existentes

Nós disponibilizamos duas listas de pseudopalavras nos Anexos. Com as listas de pseudopalavras existentes, é possível encontrar uma variedade de recursos que foram criados para utilizar na terapia, como cartões do Word para imprimir, listas de pseudopalavras no *PowerPoint* e fichas de dados para a fase prática de cada sessão. Esses recursos foram utilizados em um ou mais estudos da pesquisa.

Essas listas só serão úteis se o conjunto inteiro das consoantes e das vogais forem alvos apropriados para a criança, então, por favor use-as com cautela. As pseudopalavras estão fonologicamente e foneticamente adequadas para o português brasileiro, portanto você deverá considerar a sua própria variedade linguística e modificar também o grafema ou a transcrição.

2. Modificando listas de pseudopalavras existentes

Se você desejar modificar as listas, por favor use a função 'substituir tudo' no Word ou *PowerPoint* para se certificar de que você não perdeu nenhuma alteração. Você pode substituir o som de uma consoante ou vogal, conforme necessário, para que as pseudopalavras correspondam às consoantes que você selecionou para a criança.

3. Criando pseudopalavras manualmente.

1. Crie sílabas sem sentido

- Crie sílabas sem sentido a partir das consoantes e vogais escolhidas. Cada consoante e cada vogal deverão ser combinadas umas com as outras.

Por exemplo:

Vogal	/p/	/s/	/d/	/k/

**/u/**	/pu/	/su/	/du/	/ku/
**/a/**	/pa/	/sa/	/da/	/ka/
**/i/**	/pi/	/si/	/di/	/ki/
**/e/**	/pe/	/se/	/de/	/ke/

2. Combine as sílabas sem sentido em pseudopalavras

- Para as pseudopalavras você precisará compilar sílabas sem sentido no nível de complexidade adequado para cada criança. Crie 7 pseudopalavras no formato [fFf] (paroxítona)- Sílaba átona (sílaba fraca), sílaba tônica (sílaba Forte), sílaba átona (sílaba fraca). Por exemplo: [pe.'sa.ki]

- Crie 7 pseudopalavras no formato [ffF] (oxítona) - Sílaba átona (sílaba fraca), sílaba átona (sílaba fraca), sílaba tônica (sílaba Forte). Por exemplo: [pi.sa.'ku]

- Crie 6 pseudopalavras com o formato [Fff] (proparoxítona) -[4] Sílaba tônica (sílaba Forte), sílaba átona (sílaba fraca), sílaba átona (sílaba fraca). Por exemplo: ['pi.ke.su].

Obs: Na mesma pseudopalavra, as vogais e consoantes não devem ser repetidas.

Se a criança precisa de pseudopalavras de duas sílabas, então crie-as com o formato [Ff] e [fF], deixando de fora a sílaba final: [Ff] ['pa.ki] e [fF] [pe.'sa].

A grafia das pseudopalavras

Na pesquisa usamos principalmente palavras que seguiram o padrão ortográfico do português brasileiro. Isto significa que usamos grafias típicas que sempre são produzidas apenas de uma maneira. Nós sugerimos que você use grafias bem claras, e verifique com os adultos que fazem parte do círculo de conversa da criança como eles pronunciam as pseudopalavras, você deve checar cada palavra de acordo com a sua variedade linguística.

Preparação final para terapia

Pratique a pronúncia das pseudopalavras

Se quiser, faça a transcrição fonética das pseudopalavras para poder pronunciá-las de modo mais consistente. É importante que seus exemplos sejam consistentes e “normais”, isto é, deve-se ter uma distinção clara entre sílabas tônicas e átonas, velocidade adequada de fala e outras características prosódicas (duração da sílaba, altura e volume). **Não exagere na produção dos modelos.**

[4] [Forte-fraco-fraco] se refere à acentuação silábica.

Prepare seus materiais de terapia

Escreva ortograficamente as vinte e uma pseudopalavras no *PowerPoint*, cartões ou pedaços de papéis.

Iniciando a terapia

Modelo de Plano de Sessão

Tempo	Sessões 1-2
0 - 0.05	Boas-vindas, visão geral do paciente
0.05 - 0.25	Treinamento
0.25 - 0.55	Prática incluindo:<br>1. 20 tentativas (2 minutos de jogo)<br>2. 20 tentativas (2 minutos de jogo)<br>3. 20 tentativas (2 minutos de jogo)<br>4. 20 tentativas
0.55 (min)	Síntese, despedida

Tempo	Sessões 3-12
0 - 0.05	Boas-vindas, visão geral do paciente
0.05 - 0.15	Treinamento
0.25 - 0.55	Prática incluindo:<br>1. 20 tentativas (2 minutos de jogo)<br>2. 20 tentativas (2 minutos de jogo)<br>3. 20 tentativas (2 minutos de jogo)<br>4. 20 tentativas (2 minutos de jogo)<br>5. 20 tentativas
0.55 (min)	Síntese, despedida

Fase de treinamento

A primeira parte da sessão é destinada a desenvolver habilidades para as produções corretas das pseudopalavras que serão treinadas na fase prática, o que significa que ela deve entender a tarefa e adquirir um conhecimento prévio. Essa fase corresponde à Pré-Prática descrita na literatura da aprendizagem motora. Os passos para o ensino dos conceitos do ReST devem incluir:

1. Explicar para a criança que ela deve **pronunciar da mesma maneira que o terapeuta pronuncia as palavras.**
2. Explicar para a criança as seguintes orientações (de modo que a criança compreenda a solicitação do terapeuta):
    a) Imitar todos os fonemas - pronunciar do mesmo modo que o terapeuta.
    b) Imitar os acentos lexicais- pronunciar as sílabas tônicas corretamente.
    c) Imitar a coarticulação- ter todas as sílabas se juntando na pronúncia do mesmo modo que o terapeuta.

    Obs.: Essas três etapas precisam ocorrer ao MESMO TEMPO para que a produção seja correta, mas é possível começar a ensiná-las separadamente e depois combiná-las na fase de treinamento.

3. Selecionar aleatoriamente as pseudopalavras para o treinamento (na primeira sessão) OU selecionar, para a próxima sessão, pseudopalavras que foram produzidas consistentemente de forma incorreta nas sessões anteriores.
4. Fornecer algumas/todas as pistas para a criança indicando como ter uma pronúncia correta. Isso pode incluir:
    a) Usar exemplos de sons, de pseudopalavras, auxílios visuais e explicações, entre outros;
    b) Solicitar uma repetição para a criança do estímulo a ser trabalhado;
    c) Fornecer *feedback* de *"conhecimento dos resultados"*- dizendo se está correto ou não e porquê. Por exemplo: *"Bom trabalho, a sua pronúncia foi bem suave"* ou *"Muito bem, os sons estão bons, porém as sílabas não estão juntas umas com as outras, estão cortadas (coarticulação ineficiente). Vamos tentar de novo, mas dessa vez com todas as partes da palavra se juntando"*.
    d) Pedir uma auto-avaliação da criança;
    e) Colocar uma gravação para que a criança ouça sua própria produção de fala;
    f) Separar a pseudopalavra formada por três sílabas, transformando-as em uma de duas sílabas + uma sílaba, desenvolver a precisão da pronúncia da criança e depois recombiná-las em 3 sílabas novamente. **Tome cuidado para não pronunciar o modelo em staccato (sílabas muito curtas) ou de maneira robótica (com acentuação igualada ou prosódia achatada, sem a tonicidade destacada);**
    g) Algumas crianças são impulsivas em suas produções e podem insistir em uma produção inadequada por não conseguirem modificar o plano motor antigo. Dê dicas e explicações para que a criança adie a tentativa da produção da palavra por alguns segundos. Elas não precisam começar imediatamente e algumas se beneficiam com essa pausa;
    h) Produzir com velocidade normal ou lenta, simultaneamente, com o terapeuta;
    i) Diminuir a velocidade para aumentar a precisão, e então, acelerar novamente. Se diminuir, tenha cuidado para manter o padrão correto de acentuação lexical durante a pronúncia das sílabas.
5. Repita todos estes passos conforme necessário, até a criança fazer **5 produções corretas em QUALQUER pseudopalavra** com qualquer *feedback* ou instruções necessárias (não é necessário que sejam produções espontâneas ou consecutivas).

Essa fase pode durar de 20 a 30 minutos nas sessões 1 e 2, porém devem ser restringidas a não mais que 10 minutos em todas as sessões subsequentes. Se, por acaso, esta fase chegar aos 10 minutos em tais sessões e a criança ainda não tiver conseguido produzir cinco tentativas corretas, mesmo com várias dicas, pode ser necessário regredir para pseudopalavras formadas por duas sílabas.

Ensinando os conceitos de Batidas, Sons e Suavidade

A depender da idade da criança, utiliza-se:

- **Para crianças que são alfabetizadas**, podemos utilizar palavras escritas com os termos: Sons, Acento e Suavidade (coarticulação); Papel e lápis para desenhar forte (tônica) e fraco (átonas); Escrever letras e exemplificar com imagens.
- **Para crianças não alfabetizadas,** os terapeutas podem utilizar blocos de madeira/ tiras magnéticas de comprimentos variáveis indicando sílabas átonas e tônicas; Tambor para batidas (acentos) fortes e fracas; Um trem de brinquedo com dois vagões que se prendam ou com elos de correntes para ensinar a suavidade; Imagens que a criança já associa com sons individuais; Bonecas ou imagens de pessoas/ animais que podem ser feitos para darem as mãos (suavidade)

Fase de Prática

ESTA É A PARTE MAIS IMPORTANTE DO TRATAMENTO

Esta sessão é projetada em torno dos princípios da aprendizagem motora conhecidos por facilitar mudanças ao longo prazo em habilidades motoras.

Não invista mais tempo na fase de treinamento do que na fase prática, pois você não obterá os mesmo resultados. Para uma visão geral dos princípios da aprendizagem motora consulte *Mass et al.*, (2008).[5]

A prática acontece em 4 ou 5 blocos com 20 tentativas cada, com jogos não terapêuticos (lúdicos) de 2 minutos entre os blocos para recompensar a criança e fornecer um pequeno intervalo da prática intensa. Os jogos escolhidos devem ser motivadores para ambos (terapeuta e paciente), como basquete na lixeira, jenga ou jogo da velha, porém não deve ser focado em fala, linguagem ou escrita (Exemplo: não utilizar palavras cruzadas).

Lembre a criança que ela precisa pronunciar os sons, os acentos e a coarticulação corretamente e ao mesmo tempo para que as respostas sejam consideradas corretas. Além disso, avise-a que você vai sinalizar quando a resposta estiver certa ou errada, mas não vai poder ajudá-la a corrigir. Peça para que ela ouça cuidadosamente e apenas tente dizer a palavra quando estiver pronta.

Os arquivos de *PowerPoint* e as fichas de dados possuem as pseudopalavras apresentadas aleatoriamente. Apresente o primeiro cartão ou o slide do *PowerPoint* e diga a pseudopalavra para a criança. Peça a criança que te imite. Ouça a produção da criança e verifique se ela correspondeu ou não à sua produção (imitação). Para que seja considerada correspondente, a palavra precisa ter os mesmos fonemas, o mesmo padrão acentual e a mesma coarticulação do modelo do terapeuta.

Fluxograma da Sessão de Terapia

---

[5] Maas E., et. al. Principles of Motor Learning in Treatment of Motor Speech Disorders. American Journal of Speech-Language Pathology. 2008; 17:277–298. doi: 10.1044/1058-0360(2008/025)

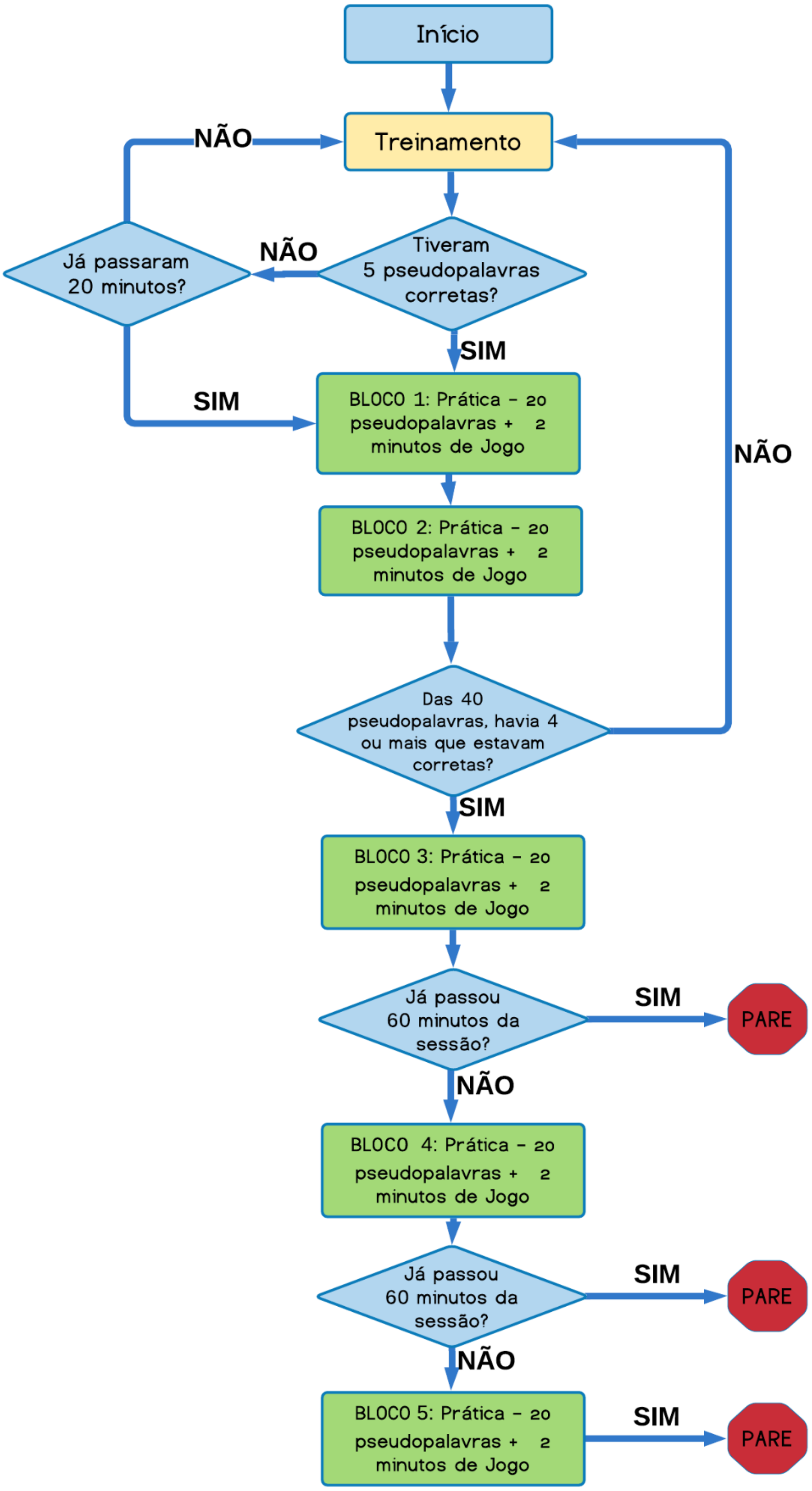

Transcreva foneticamente as respostas da criança- isso lhe dará um atraso antes de dar o *feedback* (*feedback* com atraso). Você deve aguardar por pelo menos 3 segundos para dar o

feedback. *Feedback* atrasado é um princípio da aprendizagem motora, sendo conhecido por facilitar o armazenamento da produção correta e da auto-avaliação.

O objetivo é ter 100 produções em cada sessão. Você deve apresentar 20 pseudopalavras selecionadas aleatoriamente em um bloco e, então, dar um pequeno intervalo.

Fornecer *feedback* para a criança

Feedback "Conhecimento dos resultados"

Dê um *feedback* para a criança saber se ela falou a palavra da mesma maneira que você (corretamente) ou de forma diferente (incorretamente). **Não dê instruções de como corrigi-la.** Esse tipo de *feedback* é chamado de "Conhecimento dos Resultados". Por exemplo: "Bom trabalho", "Não foi dessa vez". Ou seja, não se deve dar *feedback* específicos com detalhamentos, porém é importante apenas dar o *feedback* de conhecimento dos resultados, já que facilita para a criança a fazer mudanças auto-direcionadas à sua fala.

Você pode dar também um *feedback* geral, como "naquele bloco você acertou 10/20". Isso é motivador para crianças mais velhas.

Taxa de Feedback

Dê feedback em 50% dos itens na fase prática. **As linhas em branco da Tabela do Anexo 5 da ficha de dados indicam em quais itens o *feedback* deve ser fornecido**. As linhas em branco foram alocados aleatoriamente nos itens da seguinte maneira:

- No primeiro bloco, dê *feedback* aleatoriamente para 18 das 20 tentativas.
- No bloco seguinte, dê *feedback* para 14 das 20 tentativas.
- No terceiro bloco, dê *feedback* para 10 das 20 tentativas.
- No quarto bloco, dê *feedback* para 6 das 20 tentativas.
- E no bloco final, dê feedback para 2 das 20 tentativas.

Isso fornece *feedback* aleatório em aproximadamente 50% das tentativas. O *feedback* aleatório e de baixa frequência resulta em uma melhor retenção do conhecimento que o feedback de 100% dos itens.

Assim, há *feedback* aleatório em aproximadamente 50% das tentativas. Um *feedback* aleatório e com baixa frequência resulta em uma melhor retenção de conhecimento do que o *feedback* em 100% dos itens.

Progresso para o nível da frase

Como mencionado acima, as crianças que conseguem, com precisão, produzir pseudopalavras de três sílabas com 80% de acertos por duas sessões consecutivas devem ser avançadas para as sentenças de Cloze. São necessárias dez frases, sendo que as frases e as pseudopalavras devem ser apresentadas aleatoriamente. Isso é mais difícil, não somente porque a criança tem que

acertar todos os aspectos da pseudopalavra corretamente, mas também porque elas precisam falar a frase completa correspondendo com a sua produção de fonemas, acentos e coarticulação.

Abaixo estão as sentenças que já usamos previamente. Você irá notar que a palavra-alvo sempre é tratada como um substantivo e sempre está na posição final.

1. Eu vi um/uma ______________________.
2. Eu quero um/uma __________________.
3. Ela tem um/uma grande ________________.
4. Eu fui a/ao _________________.
5. Onde está o/a ____________________?
6. Ele me deu um/uma _______________.
7. É dele esta/este ________?
8. Tem um/uma____________
9. Pode me dar um/uma ________?
10. Aqui está o/a novo/nova ______________.

Obs: Como no português há três padrões de acentuação, você pode selecionar quatro pseudopalavras (fFf); três pseudopalavras (Fff); e três pseudopalavras (ffF) para compor as sentenças Cloze.

Qualquer frase elaborada como uma pergunta precisa ser pronunciada como uma pergunta (entonação final crescente) e se for uma afirmação, precisa ter uma prosódia de afirmação. Caso a criança obtenha 80% de acerto nas frases, você pode fazê-las mais complexas, incluindo duas pseudopalavras. Aqui está um exemplo dessa frase:

Eu vi um/uma ____________ e um/uma ______________.

Avaliando produções corretas e incorretas/Ensinando os componentes

Cada tentativa de pronúncia das pseudopalavras deve ser avaliada de acordo com a acurácia dos fonemas, acento lexical e coarticulação. Na fase prática, para que a criança pontue como acerto, todos estes aspectos precisam estar corretos simultaneamente durante a pronúncia da pseudopalavra.

Fonemas: São as consoantes e as vogais. Os sons da criança devem corresponder com o modelo do adulto, isto é, se a criança produz o som "f" distorcido, a categoria "fonemas" estará incorreta. O nosso objetivo é a produção precisa e consistente de todos os fonemas.

Acentos: Referem-se ao acento lexical entre as sílabas, sendo que a produção também precisa corresponder à do modelo adulto. Assim como no inglês, no português, para as sílabas tônicas alongamos a vogal, aumentamos o volume da sílaba ou elevamos o tom (frequência) da sílaba.

Na fase de treinamento, a criança pode se beneficiar de qualquer um desses recursos, mas descobrimos que o mais fácil é o comprimento (duração) e o mais difícil é o tom (frequência). Ao selecionar uma dica para ensinar o conceito de acento, inicie deixando a sílaba com a duração mais longa ou mais curta. Se não obter sucesso durante várias sessões, deixe a sílaba mais forte ou mais fraca, e, finalmente, dê uma pista para uma mudança no tom. Qualquer produção robótica, lenta ou que não possua acentuação deve ser considerada como incorreta. Crianças mais novas costumam ter mais problemas com as palavras estruturadas em [fFf] do que as estruturadas em [Fff], então é necessário prestar atenção neste aspecto na próxima fase de treinamento. Acentos fortes estão sinalizadas com um acento agudo ou circunflexo na sílaba tônica nos conjuntos de pseudopalavras já existentes.

Coarticulação: É o mais difícil de avaliar e exige que as sílabas sejam contínuas umas das outras. Obviamente, se você tiver uma consoante plosiva, haverá uma pequena pausa entre as sílabas, mas novamente, a produção da criança deve corresponder com a produção do adulto. Qualquer produção que não soe natural ou seja muito curta (staccato) está errada.

Se houver dúvidas sobre a pronúncia estar correta ou não, VOCÊ DEVE CONSIDERÁ-LA INCORRETA.

Acompanhando o progresso

Há duas questões principais para responder quando você está completando o tratamento da criança.

A primeira é: o tratamento está funcionando e as dicas estão fazendo a produção da criança mudar? Isso é respondido com os seus dados das sessões de tratamento. As fichas de dados (Anexo 5) das sessões podem ajudar você a responder se as produções da criança estão (1) se tornando mais precisas na pré-prática/treinamento e quais dicas lhe ajudaram, e (2) se elas estão se tornando mais precisas na prática ao longo do tempo, o que significa que elas estão aprendendo a falar pseudopalavras com o fonema, o acento lexical e a coarticulação de modo simultâneo e correto.

A segunda é: o tratamento está mudando o planejamento e a programação da criança durante a pronúncia de outras palavras reais? É isto que os familiares estão mais entusiasmados em identificar na criança e isso precisa ser mensurado. Isso é respondido por dados de generalização e deve ser planejado antes que o tratamento comece, portanto palavras similares com 2 ou 3 sílabas sozinhas e em frases são investigadas antes do tratamento, no início da sessão 4, 8 e 12 a fim de mostrar o progresso. Exemplos de sondagem de generalização que usamos estão no Anexo 6. Além disso, antes e depois do tratamento, você pode avaliar a inteligibilidade e outras avaliações específicas de fala da criança. Por exemplo, utilizamos subtestes de inconsistência de fala e avaliação de fonologia para este objetivo.

Progresso da Terapia

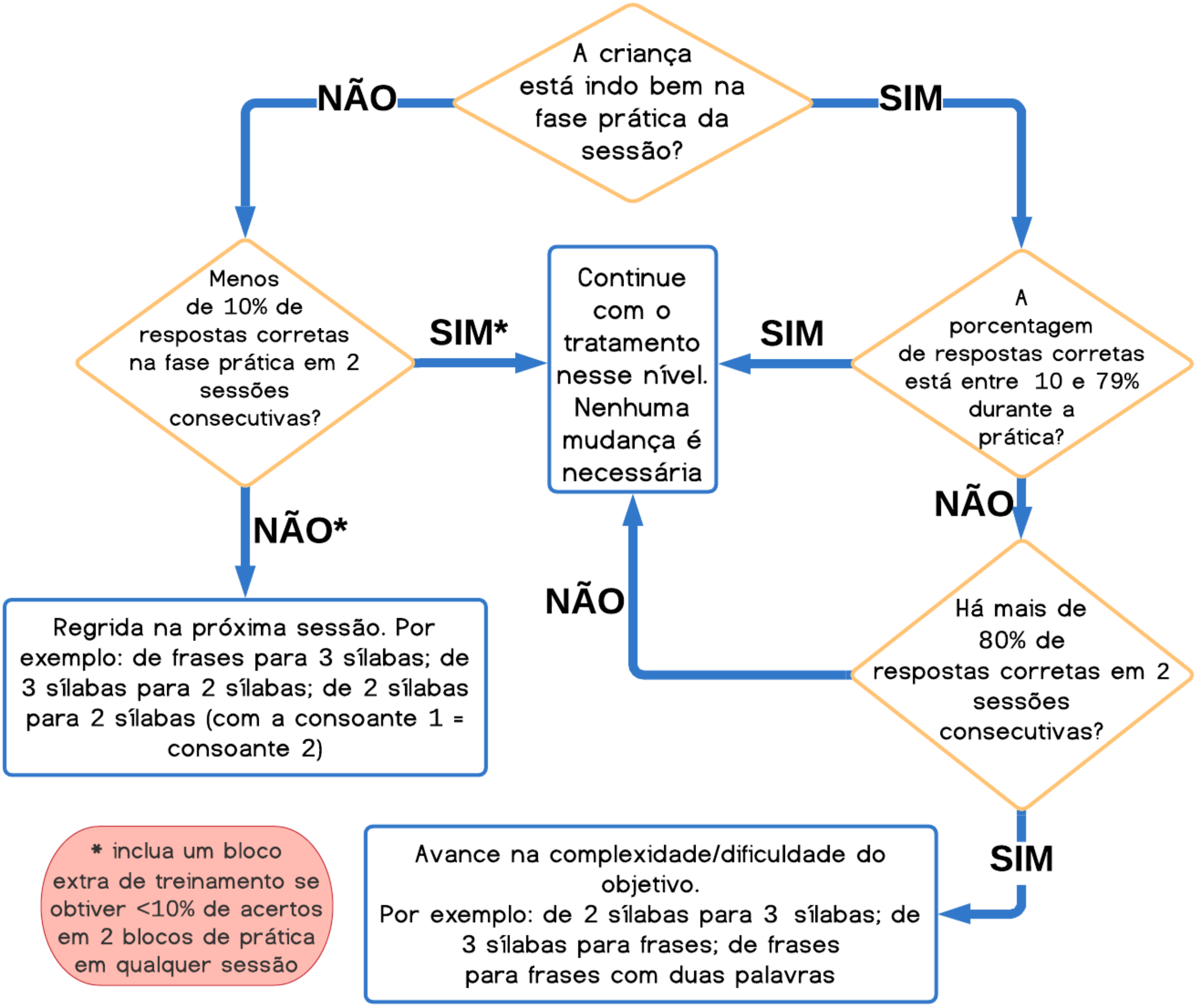
A criança está indo bem na fase prática da sessão?
NÃO
SIM
Menos de 10% de respostas corretas na fase prática em 2 sessões consecutivas?
SIM*
Continue com o tratamento nesse nível. Nenhuma mudança é necessária
SIM
A porcentagem de respostas corretas está entre 10 e 79% durante a prática?
NÃO*
NÃO
NÃO
Regrida na próxima sessão. Por exemplo: de frases para 3 sílabas; de 3 sílabas para 2 sílabas; de 2 sílabas para 2 sílabas (com a consoante 1 = consoante 2)
Há mais de 80% de respostas corretas em 2 sessões consecutivas?
* inclua um bloco extra de treinamento se obtiver <10% de acertos em 2 blocos de prática em qualquer sessão
Avance na complexidade/dificuldade do objetivo.
Por exemplo: de 2 sílabas para 3 sílabas; de 3 sílabas para frases; de frases para frases com duas palavras
SIM

ANEXO 1- Lista Trissílabas - /k/, /f/, /l/, /d/

Forte-fraco-fraco	fraco-Forte-fraco	fraco-fraco-Forte
Dákilu	Kafídu	Kefidú
Dúfila	Lefáki	Fekadí
Káfedi	Fekída	Dukilá
Fíkadu	Lidúka	Lakedí
Ládeki	Ledáki	Kufadí
Lífedu	Feládi	Lakidú
	Kufádi	Fekudá

ANEXO 2- Lista Dissílabas- /k/, /f/, /l/, /d/

Forte-fraco	fraco-Forte
Dáki	Kafí
Dúfi	Lefá
Fúki	Fekí
Fíka	Lidú
Láde	Ledá
34Lífe	Lakí
Kíla	Kufá
Káfe	Kadú
Káli	Leká
Fíka	Felá

ANEXO 3- Lista Trissílabas- /k/, /s/, /ʒ/, /l/

Forte-fraco-fraco	fraco-Forte-fraco	fraco-fraco-Forte
Lássoki	Kassíju	Kessijú
Júkila	Lojíssa	Kejadí
Sákoji	Sujíla	Lukijá
Lússika	Sakôji	Lakessí
Líkossa	Jassôki	Kujissá

Kájuli	Julássi	Jekilú
	Lajôku	Fejuká

ANEXO 4- Lista Sentenças Cloze /k/, /s/, /ʒ/, /l/

**Lista Sentenças Cloze**
1.Eu vi uma Líkossa
2.Eu quero uma Sujíla
3.Eu quero um Kissôja
4.Onde está a Lojíssa?
5.Ele me deu esse Kujissá
6.É dele esse Lássoki?
7.Tenho uma Julássi
8.Pode me dar um Kessijú?
9.Aqui está o novo Jekilú
10. Ele tem muito Kájuli

ANEXO 5- Ficha de dados das sessões

Bloco 1	Transcrição	Acento	Fonema	Coarticul	0/1	Tipo de Feedback
						CR
						CR
						CR
						CR
						CR
						CR
						CR
						CR
						CR
						CR
						CR
						CR
						CR
						CR
						CR
						CR
						CR
						CR
					/20	
2 minutos de intervalo						
Bloco 2						
						CR
						CR
						CR
						CR
						CR
						CR
						CR
						CR
						CR
						CR
						CR
						CR
						CR
						CR
					/20	

Bloco 3	Transcrição	Acento	Fonema	Coarticul	0/1	Tipo de Feedback
						CR
						CR
						CR
						CR
						CR
						CR
						CR
						CR
						CR
						CR
					/20	
2 minutos de intervalo						
Bloco 4						
						CR
						CR
						CR
						CR
						CR
						CR
					/20	

Bloco 5	Transcrição	Acento	Fonema	Coarticul	0/1	Feedback Type
						CR
						CR
						CR
					/20	

Total: /100

ANEXO 6- Exemplo de lista de sondagem

Lista de Sonda - /k/, /f/, /l/, /d/

- **20 pseudopalavras tratadas**

Forte-fraco -fraco	fraco-Forte-fraco	fraco-fraco-Forte
Dákilu	Kafídu	Kefidú
Dúfila	Lefáki	Fekadí
Káfedi	Fekída	Dukilá
Fíkadu	Lidúka	Lakedí
Ládeki	Ledáki	Kufadí
Lífedu	Feládi	Lakidú
	Kufádi	Fekudá

- **20 pseudopalavras não tratadas**

Forte-fraco-fraco	fraco-Forte-fraco	fraco-fraco-Forte
Dákeli	Lekáfi	Fikadú
Fúdila	Fedíka	Ledufí
Láfedu	Lekádu	Kelafú
Lífuka	Lafíke	Fakilê
Kádefi	Kudáfi	Delakí
Fílaku	Ledáku	Lafekú
	Fedáli	Kadelú

- **<u>20 Sentenças Cloze com pseudopalavras de 3 sílabas</u>**

1. Eu quero um **Kafídu.**
2. Ela tem uma grande **Lefáki.**
3. Eu fui ao **Fekída.**
4. Onde está o **Lidúka**?
5. Ele me deu uma **Dákilu**.
6. É dele este **Dúfila**?
7. Tenho uma **Káfedi.**
8. Pode me dar um **Kefidú** ?
9. Aqui está o novo **Fekadí.**
10. Eu vi uma **Dukilá.**
11. Ela comprou um **Lekáfi.**
12. Minha mãe tem um **Fedíka.**
13. Você viu o **Lakífe** ?
14. Ela foi ao **Lekádu.**
15. Eu chamei a **Dákeli.**
16. Ela se chama **Fúdila.**
17. Onde está o **Láfedu**
18. Eu quero uma **Fikadú.**
19. Eu fui ao **Ledufí.**
20. Tenho um **Kelafú.**

- **<u>20 Palavras reais- /K//F/ /L/ /D/- /A/ /I/ /U/ /E/</u>**

1. Cadela
2. Dedica
3. Falado (/u/)
4. Facada
5. Daquilo (/u/)
6. Ficado (/u/)
7. Falido (/u/)
8. Caído (/u/)
9. Daquele
10. Colado (/u/)
11. Café
12. Fica
13. Lado
14. Dado (/u/)
15. Cada
16. Fila
17. Quilo (/u/)
18. Fala
19. Dica
20. Cola

- 10 itens de controle (erro de articulação ou processo fonológico que hipotetizamos que não mudaria durante o tratamento ReST)

1. Chuva
2. Faixa
3. Sujo
4. Jarra
5. Zebra
6. Sujeira
7. Xícara
8. Sozinho
9. Fechado
10. Chuveiro

REFERÊNCIAS

1. McCabe P, Murray E, Thomas D, & Evans P. *Clinician Manual for Rapid Syllable Transition Treatment.* The University of Sydney, Camperdown, Australia. 2017.

2. Maas E., et. al. Principles of Motor Learning in Treatment of Motor Speech Disorders. American Journal of Speech-Language Pathology. 2008; 17:277–298. doi: 10.1044/1058-0360(2008/025)
