# Supplementary material for: Translation and cross-cultural adaptation of the therapeutic intervention manual Rapid Syllable Transition Treatment (ReST) into brazilian portuguese
Source: Codas. 2023 Mar 17;35(2):e20210257. doi: 10.1590/2317-1782/20212021257en (PMC10029998; doi:10.1590/2317-1782/20212021257en)
Supplement: Translation

# Translation: Tradução e adaptação cultural do manual de intervenção terapêutica Rapid Syllable Transition Treatment (ReST) para o português brasileiro

**DOI:** 10.1590/2317-1782/20212021257pt

**Authors:** Beatriz de Oliveira, Aline Mara de Oliveira

**Affiliations:** 1 Departamento de Fonoaudiologia, Centro de Ciências da Saúde - CCS, Universidade Federal de Santa Catarina - UFSC - Florianópolis (SC), Brasil.

**Keywords:** Apraxia, Terapia de Linguagem, Linguagem Infantil, Fonoaudiologia, Distúrbios da Fala

## Abstract

**Objetivo:**

Traduzir e adaptar culturalmente, para o português brasileiro (PB), o manual do método de intervenção *"Rapid Syllable Transition Treatment (ReST)”*.

**Método:**

O processo de tradução e adaptação cultural seguiram os critérios propostos por Beaton e Guillemin: Estágio 1 - Tradução; Estágio 2 - Síntese de Traduções; Estágio 3 - Retrotradução; Estágio 4 - Revisão por Comitê de Especialistas; Estágio 5 - Estudo-Piloto; e Estágio 6 - Avaliação e apreciação dos relatórios escritos pelo pesquisador e comitê de especialistas.

**Resultados:**

Na etapa de adaptação, foi verificada a necessidade da modificação de termos e instruções traduzidos para o português, pelos tradutores, para o contexto da prática clínica fonoaudiológica como os termos: "beats", "sounds" e "smooth", assim como a adequação da escolha das vogais, visto que no PB não há vogal schwa [ə], não sendo possível fazer uma “equivalência” para o português. O estudo-piloto também indicou necessidade de modificação no manual (Estágio 5), sendo necessário acrescentar mais um padrão de acentuação lexical, visto que no PB existem três padrões de acentuação lexical, e não dois como previsto no manual original em inglês. Atualmente na literatura são escassos os métodos de intervenção adaptados para o PB para crianças com transtornos motores de fala. Dessa maneira o manual traduzido e adaptado foi pautado na adaptação italiana do ReST, com três padrões de acentuação lexical e modificações perante a seleção das vogais, dessa maneira corroborando com o contexto linguístico do Brasil.

**Conclusão:**

O manual do *Rapid Syllable Transition Treatment (ReST)* está adaptado para o contexto linguístico do português brasileiro, sendo a intervenção no setting clínico presencial ou por meio do teleatendimento. Dessa maneira sugere-se novos estudos para comprovar a eficácia através de uma maior amostra de crianças.

## INTRODUÇÃO

As alterações de fala são decorrentes de diversas etiologias e acometem os diferentes níveis de produção da fala: nível fonológico e/ou nível motor. Sendo assim, é possível encontrar alterado, o nível fonológico, presente no desvio fonológico, e/ou o nível motor, responsável pela execução e planejamento motor da fala, observado na apraxia de fala na infância e no atraso motor de fala, ambas as alterações de produções de fala estão inseridas em um rol mais amplo, chamado transtornos dos sons da fala (TSF)^(1-3)^.

Segundo Shriberg et al.^(4,5)^ , o Sistema de Classificação dos Transtornos dos Sons da Fala é categorizado em três tipos principais de desordens da fala, sendo eles atraso de fala (desvio fonológico), erros residuais de fala e o os transtornos motores de fala, sendo o último dividido em quatro tipos: apraxia de fala na infância (AFI), disartria infantil (DI), atraso motor de fala (AMF) e AFI e DI associadas.

A apraxia de fala na infância (AFI) é definida como uma desordem, na qual a precisão e a consistência dos movimentos subjacentes à fala são prejudicados, sem a presença de déficits neuromusculares. O prejuízo ocorre no planejamento e/ou programação dos parâmetros de espaço-temporal quanto às posturas dos órgãos fonoarticulatórios e as sequências de movimentos musculares, resultando em alterações na produção dos sons da fala e na prosódia. Encontrando-se preservados, os sistemas motores e sensoriais, assim como as habilidades de compreensão e atenção^(1,2)^. A criança com apraxia de fala na infância quando se comunica, sabe quais palavras almeja emitir, porém não é capaz de realizar a programação das estruturas articulatórias em uma sequência adequada para a produção dos sons. A AFI é caracterizada por três achados clínicos diferenciais: alteração na coarticulação (transição de segmentos e de sílabas), variabilidade de erros com trocas ou substituições inconsistentes e alteração na prosódia (acentuação lexical)^(6)^.

A disartria infantil é uma desordem nos mecanismos de execução da fala, de origem neurológica, caracterizada por lentidão, fraqueza, imprecisão e incoordenação da musculatura da fala. Suas características são consistentes e estão alteradas nos níveis da voz, articulação, ressonância, respiração e musculatura. A disartria infantil também pode se manifestar associada a apraxia de fala na infância^(7)^.

O atraso motor de fala resulta de desordens na execução neuromotora, ou seja, há um atraso na maturação do sistema motor da fala, provocando alterações na precisão articulatória, na estabilidade da fala, na voz e na prosódia. Assim como também pode ser encontrado falta de dissociação dos movimentos da fala e da amplitude de movimentos excessivos não esperados para a idade. A criança necessita apresentar pelo menos quatro de doze marcadores motores de fala descritos como quatro bandeiras vermelhas para o diagnóstico de atraso motor de fala, descritos como: variedade limitada de movimentos motores da fala; produções inconsistentes; produção de poucas vogais e / ou distorções vocálicas; produção de poucas consoantes e / ou tem distorções consonantais; estruturas limitadas de sílabas e palavras; processos fonológicos inadequados para a idade; entonação atípica; tom, frequência, volume e nasalidade inadequados; dificuldade em manter a integridade do som e da sílaba com aumento do comprimento e complexidade do enunciado; aumento da variabilidade de erros; tateio articulatório; fadiga e ininteligibilidade de fala^(5,8,9)^.

As intervenções fonoaudiológicas voltadas para esse grupo de crianças encontradas na literatura, estão associadas ao tratamento intensivo, o qual tem sido recomendado para o tratamento dos sons da fala, especificamente dos transtornos motores de fala^(10)^. A terapia intensiva consiste na aplicação dos princípios da Aprendizagem Motora, descritos como: tipo de feedback- feedback de Informação de Desempenho (*“faltou abrir aquele sorriso”*) ou Informação de Resultado (IR, *“correto”*, *“quase certo”*); frequência de *feedback*- alta frequência durante a fase de pré-prática e diminuição gradual durante a fase prática; variabilidade de prática- variar parâmetros como o ritmo, a velocidade de fala, a intensidade, o timbre e o tom vocal; e o aumento da complexidade do estímulo com base no desempenho da criança^(11)^.

A escassez de metodologias de tratamento fonoaudiológico voltadas para o tratamento de crianças com transtornos motores de fala, justifica-se a elaboração e/ou a adaptação de modelos de intervenções que estejam ancoradas nos critérios linguísticos do português brasileiro, o que espera-se maior efetividade no tratamento.

O *Rapid Syllable Transition Training* (ReST)^(12-15)^ foi projetado para abordar diretamente os problemas de planejamento e de programação motora subjacentes à fala, experimentados por crianças australianas com Apraxia de Fala na Infância, particularmente com o gerenciamento do acento lexical e a transição da sequência de segmentos ou de sílaba para sílaba. Com o objetivo de trabalhar os princípios de aprendizagem motora (neuroplasticidade), para que cada sessão de tratamento fosse projetada para ter duas partes: primeiro uma fase de Treinamento (chamada de pré-prática na literatura de aprendizagem motora) e segundo uma fase de Prática (também chamada de prática na literatura de aprendizagem motora).

O tratamento com ReST^(12-15)^ consiste na prática de alta intensidade de vinte pseudopalavras apresentadas aleatoriamente, com variações na estrutura fonética e no acento lexical. As vinte pseudopalavras são apresentadas em quatro a cinco blocos na fase prática, totalizando cem produções em cada sessão. O uso de pseudopalavras permite que as crianças pratiquem o planejamento e a programação motoras em formas semelhantes às palavras reais, sem interferência de planos previamente aprendidos incorretamente. O ReST é projetado para ativar e para usar componentes do sistema motor da fala sem referência ao sistema de linguagem mais amplo (exceto o subsistema Fonologia), por meio de pseudopalavras elencadas para cada criança. O objetivo é criar novos planos motores, praticá-los com frequência e usar *feedforward* (orientação para produções futuras) e *feedback* (resposta imediata ao desempenho da produção da criança) para melhorar a produção das pseudopalavras, vislumbrando melhorar a habilidade da criança de usar o planejamento e a programação da fala de forma eficaz.

Atualmente, o método de intervenção ReST está disponível para o inglês australiano, o qual foi desenvolvido originalmente para atender a demanda de crianças australianas com AFI, assim como, mais recentemente foi adaptado para a língua italiana^(16)^.

Entretanto, para estimular a disseminação do tratamento, os autores^(12-15)^ explicitam em seu site a liberdade do desenvolvimentos de recursos para ReST em demais línguas por outros fonoaudiólogos de outros países.

A terapia com ReST também mostrou-se eficaz na modalidade de teleatendimento. Foi realizado um estudo com cinco crianças de 5 a 11 anos com diagnóstico de AFI, as quais foram submetidas a intervenção terapêutica ReST por meio de videoconferência por doze sessões (quatro vezes na semana por três semanas). Os resultados mostraram aquisição significativa da imitação de pseudo-palavras selecionadas e generalização do efeito do tratamento para pseudopalavras não tratadas e palavras reais. Esses resultados sugerem que o teleatendimento com a terapia ReST como um método de intervenção pode ser benéfico para crianças com AFI^(17)^.

Dessa forma o objetivo desse estudo foi traduzir e adaptar culturalmente, para o português brasileiro (PB), o manual de intervenção *Rapid Syllable Transition Treatment (ReST)* ^(15)^.

## MÉTODO

A presente pesquisa foi aprovada pelo Comitê de Ética em Pesquisa da Universidade Federal de Santa Catarina (CAAE: 35360620.9.0000.0121 e parecer nº 4.279.198/2020). Os responsáveis das crianças incluídas neste estudo assinaram um Termo de Consentimento Livre e Esclarecido (TCLE). A versão original do manual do método de intervenção *Rapid Syllable Transition Treatment (Rest)* foi traduzida para o português brasileiro, com o consenso e aprovação dos autores^(12-15)^ originais do método.

### Tradução e adaptação cultural

Para realizar a adaptação cultural do manual do método de intervenção ReST, foram adotados os critérios propostos por Beaton et al.^(18)^: Estágio 1 - Tradução; Estágio 2 - Síntese; Estágio 3 - Retrotradução; Estágio 4 - Revisão por Comitê de Especialistas; Estágio 5 - Estudo-piloto; e Estágio 6 - Avaliação e apreciação dos relatórios escritos. A descrição de cada estágio teve por referência Beaton et al.^(18)^ e Guillemin et al.^(19)^.

1. Tradução (estágio 1)

O manual original foi traduzido para a língua-alvo, o português brasileiro através de três tradutores (Tradutor 1 - T1; Tradutor 2 - T2 e Tradutor 3- T3). Beaton et al.^(18)^ recomendam que sejam realizadas pelo menos duas traduções e que cada tradutor detalhe, por escrito, as etapas da tradução, as dúvidas e as dificuldades enfrentadas. Ao menos um tradutor deve ter conhecimento dos objetivos do procedimento e estar familiarizado com a linguagem acadêmica, enquanto os demais não devem ter conhecimento do objetivo do procedimento ou conhecer o tema. Esse segundo profissional é chamado, por Beaton et al.^(18)^, de “tradutor ingênuo”, pois, por não ser influenciado pelo meio acadêmico, oferecerá uma tradução capaz de refletir a linguagem utilizada pela população-alvo, muitas vezes destacando significados ambíguos do procedimento original.

Essa etapa, portanto, foi realizada por três tradutores, nativos do Brasil, sendo dois moradores da região do nordeste brasileiro e um da região do sul do Brasil, com domínio das duas línguas, Inglês e Português. Dois tradutores são graduandos bilíngues de Letras-Inglês, que já atuam na área de tradução, enquanto o outro tradutor é Fonoaudiólogo especialista na área de linguagem, sobretudo na área de transtornos motores da fala. Somente a esse último tradutor foi explicado o objetivo do procedimento.

Aos tradutores, foi solicitado que a versão do instrumento para a língua portuguesa fosse realizada de forma independente, preservando a equivalência semântica de todos os itens do procedimento original.

1. Síntese das traduções (estágio 2)

De acordo com Beaton et al.^(18)^ esse estágio refere-se à síntese das traduções em uma única versão, sendo necessário que os tradutores entrem em consenso sobre as suas divergências. Para a obtenção da versão de consenso, os três tradutores se reuniram remotamente com as pesquisadoras as quais acompanharam o processo para a elaboração dessa versão, auxiliando os tradutores nas eventuais dúvidas. Foram consideradas as variações linguísticas do PB (adaptações linguísticas ver no Quadro 1). A variação fonológica não é um impeditivo para a adaptação linguística do instrumento, uma vez que acredita-se que não seja possível contemplar todas as variedades do PB presentes no Brasil, sendo necessário, adaptações do próprio clínico para adaptações de segmentos que julgarem necessários na composição das pseudopalavras. Ao final desta etapa, obteve-se a primeira versão da tradução do procedimento.

**Quadro 1:** Comparação do manual original, tradução literal e a adaptação cultural feita perante a termos e instruções do manual original a fim de se adequar à realidade linguística e cultural do Português Brasileiro

**Manual original**	**Tradução literal**	**Adaptação cultural**
Sounds	Sons	Fonemas
Beats	Batidas	Acento lexical
Smooth	Suavidade	Coarticulação
1. Choose **three long vowels** from the child’s repertoire.	1. Escolha **três vogais longas** do repertório da criança.	**Escolha 4 vogais do repertório da criança.**
2. Also select **one weak or neutral vowel (e.g. schwa).**	2. Escolha também **uma vogal fraca ou neutra** (ex: schwa).	Lembrando que caso o terapeuta escolha /e/ ou /o/ em posição de sílaba átona final, em algumas regiões brasileiras, será pronunciado como [I] e [U], respectivamente
3. For some children in the research we selected **two long, one short and schwa.**	3. Para algumas crianças da pesquisa nós selecionamos **duas vogais longas, uma curta e schwa.**	Sugere-se que o terapeuta selecione as vogais /a/, /i/, /u/ em decorrência das oposições (extremidades do triângulo vocálico) dos gestos articulatórios envolvendo as três vogais.

Em negrito, refere-se às palavras que foram traduzidas e adaptadas

1. Retrotradução (estágio 3)

Finalizadas as adaptações culturais necessárias, uma tradutora nativa dos Estados Unidos realizou a retrotradução (português - inglês). A tradutora não foi a mesma da primeira etapa e não possuía conhecimento dos objetivos do procedimento, dessa maneira trabalhou na retrotradução da versão para o inglês sem ter acesso ao original.

A finalidade dessa etapa foi verificar se a versão refletia o conteúdo da versão original. Em seguida, a versão original e a retrotradução foram comparadas e corrigidas para que os erros de tradução e de interpretações fossem excluídos. E para verificar se a versão adaptada não apresentou mudança de significância do conteúdo original.

1. Revisão por comitê de especialistas (estágio 4)

Nesse estágio, de acordo com Beaton et al.^(18)^, deve-se chegar a um consenso, a partir de todas as versões das traduções produzidas, de modo que seja elaborada uma versão pré-final.

Os materiais disponibilizados ao comitê de especialistas foram o manual original em inglês, as traduções e a síntese das traduções. Esse estágio tem por objetivo realizar as mudanças necessárias no manual de intervenção para garantir tanto a equivalência cultural quanto a semântica, idiomática e conceitual.

O trabalho de equivalência semântica, cultural, idiomática e conceitual foi realizado com a ajuda de duas pós-doutorandas em linguística. Nesse estágio, as pesquisadoras do estudo se reuniram remotamente com o comitê de especialistas para analisar o procedimento original e todas as versões produzidas para, assim, elaborar uma versão Pré-Final.

1. Aplicação da versão pré-final do manual- estudo-piloto (estágio 5)

Estudo-piloto com a finalidade de detectar possíveis erros, avaliando a qualidade da adaptação e os aspectos práticos de sua aplicação. Esse estágio permite refinar a escrita do manual (Material Suplementar), assim como a sua adaptação cultural e validação. Os participantes do estudo-piloto são dois pacientes da Clínica Escola de Fonoaudiologia da Universidade Federal de Santa Catarina, os quais participam do Projeto de Pesquisa Desvio Fonológico e Apraxia (CAAE: 35360620.9.0000.0121 e parecer nº 4.279.198/2020).

Adotaram-se os seguintes critérios de inclusão: 1) ter sido atendido previamente na Universidade Federal de Santa Catarina; 2) apresentar diagnóstico de transtornos dos sons da fala de grau severo; sem comorbidades associadas e sem perdas auditivas; 3) assinatura do Termo de Consentimento Livre e Esclarecido pelos pais ou responsáveis; 4) assinatura do Termo de Assentimento. Foram excluídos: 1) indivíduos que enquadraram como transtornos dos sons da fala de grau leve; 2) pacientes com comorbidades associadas como deficiência intelectual, síndromes ou perdas auditivas. Essas informações foram observadas no prontuário do paciente ou obtidas em anamnese.

Inicialmente, foram realizadas as avaliações fonoaudiológicas: Avaliação Fonológica a partir do instrumento de Avaliação Fonológica da Criança^(20)^. E as avaliações específicas da produção motora da fala: Avaliação de Palavras Multissilábicas; Avaliação do Acento Frasal; Avaliação da Inconsistência de fala e Avaliação Motora de Fala^(21)^. Em seguida, foi implementada a aplicação do Método de Intervenção ReST nos dois sujeitos por meio de teleatendimento.

A versão do manual adaptado para o português do método de intervenção ReST^(12-15)^ foi aplicada por doze sessões intensivas de teleatendimento em dois irmãos com transtornos dos sons da fala respectivamente: S1- criança de 9 anos com diagnóstico de atraso motor de fala e S2- adolescente de 16 anos com diagnóstico de apraxia de fala na infância. Os pacientes foram reavaliados uma semana e um mês após o término das sessões de intervenção através do instrumento de Avaliação Fonológica da Criança e cálculo da Porcentagem de Consoantes Correta (PCC-R)^(20)^.

Avaliação e apreciação de todos os relatórios fornecidos pelo pesquisador e comitê de especialistas (estágio 6)

Todas as traduções e retrotraduções bem como o material utilizado para o estudo-piloto e o material com as correções realizadas após essa etapa foram enviados para processo de auditoria com o objetivo de verificar se todas as etapas do processo de tradução e adaptação cultural haviam sido seguidas. Após este estágio, não foi permitido alterar o conteúdo do procedimento.

Dois pesquisadores Fonoaudiólogos na área de Linguagem foram convidados a participar do processo de auditoria, juntamente com os fonoaudiólogos especialistas, e foram orientados sobre os propósitos da pesquisa. Foi-lhes solicitado que indicassem se cada etapa do processo de tradução e de adaptação cultural tinha sido seguida e se o procedimento, mediante as informações entregues e estudo-piloto, estava adequado para a tradução e adaptação cultural do manual de intervenção terapêutica *Rapid Syllable Transition Treatment (ReST)* ^(12-15)^ para o português brasileiro.

## RESULTADOS

Estágio 1 (tradução) e estágio 2 (síntese das traduções)

Três tradutores fizeram as traduções separadamente, sem se comunicarem. As decisões referentes aos termos utilizados e à melhor estrutura assim como as dificuldades dos tradutores perante a versão em português foram descritas em relatório para as pesquisadoras deste estudo. No Estágio 2, os tradutores se reuniram com as pesquisadoras remotamente e realizaram uma versão única das três traduções (síntese das traduções). Os tradutores alcançaram o consenso da escrita do manual em normas ortográficas do português brasileiro, entretanto foi encontrado certas dificuldades no processo.

Os tradutores "ingênuos", sem conhecimento perante ao método de intervenção e a área de estudo, relataram dificuldade no processo de tradução devido aos termos pertencentes à área da Fonoaudiologia e da Fonética e Fonologia. Ao traduzir, foi necessário pesquisas quanto aos termos equivalentes em português que fossem pertinentes ao termo oficial.

Outra dificuldade encontrada pelos tradutores foi a tradução da fase de prática do manual, na qual os fonemas eram pertencentes a língua inglesa como a vogal schwa [ə], não sendo possível fazer uma “equivalência” para o português sem a ajuda de um fonoaudiólogo e especialista na área durante essa etapa.

Já a tradutora bilíngue fonoaudióloga relatou a dificuldade em alguns termos que foram utilizados no inglês os quais não apresentavam palavras correspondentes no português que representasse os mesmos termos fonoaudiológicos utilizados no Brasil, como por exemplo os termos *"sounds", "beats"* e *"smooth"* (Quadro 1).

Juntamente com as pesquisadoras, optou-se por modificar alguns termos e instruções traduzidos para o português pelos tradutores ao contexto da linguagem utilizada na prática clínica fonoaudiológica. Dessa maneira foi realizada a adaptação cultural do manual como exposto no Quadro 1.

Exemplos de pseudopalavras, frases e listas já prontas também foram necessários serem modificados durante a adaptação, tendo em vista que não poderiam ser aproveitadas pois não faziam parte do contexto linguístico do portugûes brasileiro, portanto foram substituídas no manual por exemplos adequados ao português brasileiro.

Após finalizar a versão única, as pesquisadoras realizaram as adequações das instruções, frases e textos para facilitar a compreensão do manual. Finalizada essa etapa, prosseguiu-se para o estágio 3 - retrotradução.

Estágio 3 (retrotradução)

Nessa etapa, após as adaptações culturais necessárias, uma quarta tradutora nativa da língua inglesa realizou a retrotradução (português - inglês), dessa maneira verificou-se que as instruções e textos do manual traduzido e adaptado para o portugês brasileiro refletiam o conteúdo da versão original. Divergindo do original apenas as etapas modificadas durante a adaptação como demonstrado acima, porém não implicaram na totalidade da significância do conteúdo original.

Estágio 4 (revisão por comitê de especialistas)

Nesse estágio, as pesquisadoras do estudo se reuniram remotamente com o comitê de especialistas para analisar o manual original e todas as versões produzidas para, assim, elaborar uma versão Pré-Final. Sendo assim os juízes concordaram que as instruções e textos refletem o conteúdo da versão original, assim como as adaptações propostas nas etapas anteriores demonstraram estar adequadas ao contexto linguístico brasileiro e suprem a demanda de adaptação cultural para por fim ser aplicado em crianças com transtornos dos sons da fala.

Estágio 5 (aplicação da versão pré-final do manual - estudo-piloto)

O estudo-piloto realizado com dois pacientes demonstrou a necessidade de modificação no manual com a adição de mais um padrão de acentuação lexical a fim de corroborar com a frequência de acentos lexicais presentes no português brasileiro, portanto foi consultada a versão da adaptação em italiano^(16)^ por orientação dos autores^(12-15)^ do manual original para realização da adaptação para o português brasileiro, dessa maneira a composição das vinte pseudopalavras foi organizada de acordo com a Tabela 1.

**Tabela 1:** Adaptações culturais dos padrões de acentuação lexical

**Manual original**	**Tradução literal**	**Adaptação cultural**
**Create 10 words with the format of [Sww]-** Strong syllable (long vowel), Weak syllable [schwa or short vowel], Weak syllable [schwa/short vowel]	**Crie dez pseudopalavras com o formato [Fff]** - sílaba Tônica (vogal longa), sílaba Átona (schwa/vogal curta), sílaba Átona (schwa/vogal curta)	**Crie 7 pseudopalavras no formato [fraco-Forte-fraco]** (paroxítona)
		**Crie 7 pseudopalavras no formato [fraco-fraco-Forte]** (oxítona)
**Create 10 words with the format of [wSw]** -Weak syllable [schwa], Strong syllable (long vowel), Weak syllable [schwa/short vowel]	**Crie dez pseudopalavras com o formato [fFf]** -sílaba Átona (schwa), sílaba Tônica (vogal longa), sílaba Átona (schwa/vogal longa)	**Crie 6 pseudopalavras com o formato [Fff]** (proparóxitona)

Em negrito refere-se às palavras que foram traduzidas e adaptadas

No estudo os sujeitos apresentaram boa aceitação à intervenção terapêutica durante as doze sessões de teleatendimento e não houve queixas ou relatos de dificuldade quanto à compreensão das instruções da aplicação do manual. As descrições de casos e a verificação da eficácia do método serão discutidos em publicações futuras, entretanto, foi possível observar bom desempenho durante as sessões (Figuras 1, 2, 3 e 4). Os dois sujeitos apresentaram avanço no nível de complexidade das pseudopalavras, pois alcançaram 80% de acertos em duas sessões consecutivas.

**Figura 1:** Desempenho do treino de dissílabas do S1 com pseudopalavras compostas por /d/, /k/, /l/, /f/, /a/, /i/, /u/, /e/

**Figura 2:** Desempenho do treino de trissílabas do S1 com pseudopalavras compostas por /d/, /k/, /l/, /f/, /a/, /i/, /u/, /e/

**Figura 3:** Desempenho do treino de trissílabas do S2 com pseudopalavras compostas por /k/, /l/, /s/, /ʒ/ /a/, /i/, /u/, /e/

**Figura 4:** Desempenho do treino de sentenças Cloze do S2 com pseudopalavras compostas por /k/, /l/, /s/, /ʒ/ /a/, /i/, /u/, /e/

Na Figura 1 é possível verificar o desempenho do treino de dissílabas do S1 com pseudopalavras compostas por /d/, /k/, /l/, /f/, /a/, /i/, /u/, /e/. Apresentando ganho de 70% na produção geral em comparação a primeira sessão ao final da sexta sessão, 77% de ganho em desempenho de fonemas, 77% de melhora na produção de acentos lexicais e 68% na produção de coarticulação. A partir da sétima sessão, S1 já havia obtido bom desempenho e aderência a terapia, dessa maneira avançou para o nível de trissílabas. Durante as sessões de intervenção, apresentou ganho de 32% na produção geral ao final da última sessão, 20% de ganho em desempenho de fonemas, 33% de melhora na produção de acentos lexicais e 20% na produção de coarticulação como exposto na Figura 2.

Quanto ao desempenho de S2 com pseudopalavras compostas por /k/, /l/, /s/, /ʒ/ /a/, /i/, /u/, /e/ é possível verificar na Figura 3 um ganho de 75% na produção geral em comparação a primeira sessão ao final da sétima sessão, 69% de ganho em desempenho de fonemas, 58% de melhora na produção de acentos lexicais e 71% na produção de coarticulação. A partir da oitava sessão S2 já havia obtido bom desempenho e aderência a terapia, dessa forma também avançou de nível como demonstrado na Figura 4. Nas sentenças cloze foi possível verificar ganho de 10% na produção geral em comparação a oitava sessão ao final da décima segunda sessão, 8% de melhora na produção de acentos lexicais, 13% na produção de coarticulação e perseverando a mesma porcentagem no desempenho de fonemas em comparação a oitava sessão ao final da décima segunda sessão.

Já no Quadro 2 é possível analisar bom prognóstico durante as reavaliações de pré-terapia e após uma semana e um mês de intervenção terapêutica respectivamente. Ressalta-se que os casos serão aprofundados em publicações futuras.

**Quadro 2:** Análise dos sujeitos em pré terapia e após doze sessões de intervenção terapêutica

**SUJEITOS**	**INSTRUMENTOS DE AVALIAÇÃO**	**PRÉ TERAPIA**	**PÓS TERAPIA - 1 SEMANA**	**PÓS TERAPIA - 1 MÊS**
	**PCC-R**	**46,56%**	**50,88%**	**48,23%**
		OI- ADQUIRIDOS	OI-ADQUIRIDOS	OI-ADQUIRIDOS /p/, /b/, /t/, /d, /g/, /v/, /m/, /n/, /dƷ/, /tʃ/ OI- PARCIALMENTE ADQUIRIDOS **/k/**
		/p/, /b/, /t/, /d, /g/, /v/, /m/, /n/, /dƷ/, /tʃ/	/p/, /b/, /t/, /d, /g/, /v/, /m/, /n/, /dƷ/, /tʃ/	
		OI- NÃO ADQUIRIDOS	OI- PARCIALMENTE ADQUIRIDOS	
		/k/, /f/, /l/, /s/, /z/, /Ʒ/, /ʃ/, /R/	**/k/, /f/, /l/**	
**S1**	**INVENTÁRIO FONOLÓGICO - (Avaliação Fonológica, YAVAS)**	OM- ADQUIRIDOS	OM- ADQUIRIDOS	OM- ADQUIRIDOS /p, /b/, /t/, /d/, /g/, /v/, /m/, /n/, /dƷ/, /tʃ/, /ɲ/ OM- PARCIALMENTE ADQUIRIDO **/k/**
		/p, /b/, /t/, /d/, /g/, /v/, /m/, /n/, /dƷ/, /tʃ/, /ɲ/	/p, /b/, /t/, /d/, /g/, /v/, /m/, /n/, /dƷ/, /tʃ/, /ɲ/	
		OM- NÃO ADQUIRIDOS	OM- PARCIALMENTE ADQUIRIDO	
		/k/, /ɾ/, /l/, /f/, /s/, /z/, /R/, /Ʒ/, /ʃ/, /ʎ/	**/k/**	
		CM- ADQUIRIDOS	CM- ADQUIRIDOS	CM- ADQUIRIDOS
		/m/, /n/,	/m/, /n/,	/m/, /n/,
		
		CM- NÃO ADQUIRIDOS	CM- NÃO ADQUIRIDOS	CM- NÃO ADQUIRIDOS
		/s/, /ʃ/, /Ʒ/, /R/	/s/, /ʃ/, /Ʒ/, /R/	/s/, /ʃ/, /Ʒ/, /R/
		
		CF- ADQUIRIDOS	CF- ADQUIRIDOS	CF- ADQUIRIDOS
		/m/, /n/, /ʃ/, /R/	/m/, /n/, /ʃ/, /R/	/m/, /n/, /ʃ/, /R/
		
		CF- NÃO ADQUIRIDOS	CF- NÃO ADQUIRIDOS	CF- NÃO ADQUIRIDOS
		/s/, /Ʒ/	/s/, /Ʒ/	/s/, /Ʒ/
	**PCC-R**	**66,28%**	**82,37%**	**81,18%**
**S2**		OI- ADQUIRIDOS	OI-ADQUIRIDOS	OI-ADQUIRIDOS
		/p/, /b/, /t/, /d, /g/, /v/, /m/, /n/, /dƷ/, /tʃ/ , /l/, /R/	/p/, /b/, /t/, /d, /g/, /v/, /m/, /n/, /dƷ/, /tʃ/ , /l/, /R/,	/p/, /b/, /t/, /d, /g/, /v/, /m/, /n/, /dƷ/, /tʃ/ , /l/, /R/,
		OI- PARCIALMENTE ADQUIRIDOS	**/k/, /Ʒ/, /s/, /f/**	**/k/, /Ʒ/, /s/, /f/**
		/k/		OI- PARCIALMENTE ADQUIRIDOS
				**/z/**
		OI- NÃO ADQUIRIDOS	OI- NÃO ADQUIRIDOS	OI- NÃO ADQUIRIDOS
		/f/, /s/, /z/, /Ʒ/, /ʃ/	/ʃ/ , /z/	/ʃ/
	**INVENTÁRIO FONOLÓGICO - (Avaliação Fonológica, YAVAS)**	OM- ADQUIRIDOS	OM- ADQUIRIDOS	OM- ADQUIRIDOS
		/p/, /b/, /t/, /d, /g/, /v/, /m/, /n/, /ɲ/, /dƷ/, /tʃ/ , /l/, /R/, /ɾ/, /ʎ/	/p/, /b/, /t/, /d, /g/, /v/, /m/, /n/, /ɲ/, /dƷ/, /tʃ/ , /l/, /R/, /ɾ/, /ʎ/,	/p/, /b/, /t/, /d, /g/, /v/, /m/, /n/, /ɲ/, /dƷ/, /tʃ/ , /l/, /R/, /ɾ/, /ʎ/,
		OM- PARCIALMENTE ADQUIRIDOS	**/f/, /k/, /Ʒ/, /s/**	**/f/, /k/, /Ʒ/, /s/**
		/k/		OM- PARCIALMENTE ADQUIRIDO
				**/z/**
		OM- NÃO ADQUIRIDOS	OM- NÃO ADQUIRIDOS	OM- NÃO ADQUIRIDOS
		/f/, /s/, /z/, /Ʒ/, /ʃ/	/z/, /ʃ/	/ʃ/
		CM- ADQUIRIDOS	CM- ADQUIRIDOS	CM- ADQUIRIDOS
		/m/, /n/	/m/, /n/, **/s/, /ʃ/**	/m/, /n/, **/s/, /ʃ/**
		CM- NÃO ADQUIRIDOS	CM- NÃO ADQUIRIDOS	CM- NÃO ADQUIRIDOS
		/s/, /ʃ/, /R/	/R/	/R/
		CF- ADQUIRIDOS	CF- ADQUIRIDOS	CF- ADQUIRIDOS
		/m/, /n/, /ʃ/, /R/, /s/	/m/, /n/, /ʃ/, /R/, /s/	/m/, /n/, /ʃ/, /R/, /s/

**Legenda:** OI- Onset Inicial; OM- Onset Medial; CM- Coda Medial; CF- Coda Final. Em negrito se encontram os fonemas adquiridos parcialmente e/ou totalmente durante as doze sessões de intervenção por S1 e S2. Os dois sujeitos não apresentam encontro consonantal em seu inventário fonológico.

Estágio 6 (Avaliação e apreciação de todos os relatórios fornecidos pelo pesquisador e comitê de especialistas):

Com a finalização das traduções, da retrotradução, do estudo-piloto e das adequações necessárias indicadas no estudo-piloto, alcançou-se a guisa da versão final do manual. Dois pesquisadores da Fonoaudiologia na área de Linguagem participaram do processo de auditoria, juntamente com os fonoaudiólogos (doutores e mestres) que compuseram a comissão de especialistas e os pesquisadores deste artigo para verificar se as etapas do processo de tradução e adaptação haviam sido seguidas e cumpridos todos os critérios.

O processo de auditoria concluiu que todas as etapas foram cumpridas e que o procedimento estava apto a prosseguir para a fase de caracterização da população.

## DISCUSSÃO

O objetivo dessa pesquisa foi traduzir e adaptar culturalmente, para o português brasileiro (PB), o manual de intervenção *"ReST- Rapid Syllable Transition Treatment”*, voltado para o tratamento de crianças com apraxia de fala na infância.

Na língua inglesa, o padrão acentual lexical em palavras polissilábicas é predominantemente posicionado na sílaba antepenúltima para substantivos (Forte-fraco-fraco), na sílaba antepenúltima e penúltima para verbos (Forte-fraco-fraco ou fraco-Forte-fraco)^(22)^. Dessa maneira, no manual original, o método de intervenção terapêutica ReST^(12-15)^, em inglês, foi implementado utilizando apenas dois padrões lexicais na língua, totalizando vinte pseudopalavras com duas variações de acentuação lexical.

Em contrapartida, assim como no português brasileiro, a língua italiana apresenta três padrões de acentuação lexicais (fraco-Forte-fraco; fraco-fraco-Forte; Forte-fraco-fraco), dessa maneira, na adaptação italiana os autores implementaram a representação dos três padrões lexicais nas pseudopalavras como estímulos da terapia^(16)^. Ao invés de aumentar o número de pseudopalavras, os três padrões lexicais foram divididos de acordo com a sua frequência na língua. Ao passo que no italiano, mais de 80% das palavras de pelo menos três sílabas recebem acentuação na penúltima sílaba (fraco-Forte-fraco - paroxítonas); 16% possuem acentuação lexical na antepenúltima sílaba (Forte-fraco-fraco- proparoxítonas) e 4% possuem acentuação na última sílaba da palavra (fraco-fraco-Forte- oxítona)^(23)^.

Por orientação dos autores^(12-15)^ originais, a etapa da adaptação cultural (Estágio 5) para o PB foi também pautada na adaptação do italiano^(16)^, dessa forma, através da análise dos padrões de acentuação lexical no português: palavras com pelo menos três sílabas com acentuação na penúltima sílaba equivalem a cerca de 62% (fraco-Forte-fraco - paroxítonas); acentuação na última sílaba estão presente em 25% (fraco-fraco-Forte- oxítona) e as menos comuns possuem acentuação lexical na antepenúltima sílaba (Forte-fraco-fraco- proparoxítonas) compõem 12%^(24)^. Sendo assim os autores, durante a adaptação optaram por compor as 20 pseudopalavras organizadas em 7 pseudopalavras no formato [fraco-Forte-fraco] (paroxítona); 7 pseudopalavras no formato [fraco-fraco-Forte] (oxítona) e 6 pseudopalavras com o formato [Forte-fraco-fraco] (proparoxítona).

Quanto às consoantes que compõem as pseudopalavras, não foi necessário adaptação em relação ao manual original^(15)^, permanecendo a instrução da seleção de quatro consoantes existentes no inventário fonológico da criança. Com relação às vogais, como o *schwa* /ə/ não é utilizado para indicar sílaba átona/ fraca no PB, optou-se por não incluir na lista de estímulo de pseudopalavras, diferente do previsto no manual na língua inglesa^(15)^. Foram inseridas as vogais orais do PB, [a], [e], [ɛ], [i], [o], [ɔ] e [u], sendo que preferencialmente, a escolha dos alvos deverá ser iniciadas pelas as vogais [a], [i] e [u] e, posteriormente, deverão ser inseridas nas pseudopalavras as vogais mediais [e], [ɛ], [o] e [ɔ].

A organização das sessões, de acordo com o método original em inglês,^(12-15)^ propõe uma fase de pré-prática composta pelo treinamento das pseudopalavras utilizando-se de qualquer pista ou feedback, e uma fase prática com quatro a cinco blocos de vinte pseudopalavras cada sem feedbacks específicos de correção, com jogos lúdicos de dois minutos nos intervalos entre blocos. Na aplicação do estágio 5 a organização das sessões mostrou-se eficaz durante as intervenções terapêuticas nas crianças do estudo-piloto. Sendo assim, a adaptação do método para o português brasileiro foi pautada no método de intervenção terapêutica em inglês e italiano^(16)^, não sendo necessário mudanças em relação a esses aspectos.

Estudos^(25,26)^ apontam a eficácia das terapias intensivas nos transtornos motores de fala, dessa forma Murray et al.^(25)^ demonstra a comparação entre dois modelos de intervenção ReST e NDP3 the Nuffield Dyspraxia Programme-Third Edition (NDP3) e apesar de ambos mostrarem fortes evidências no tratamento e generalização, ReST apresentou maiores ganhos nas crianças com apraxia de fala na infância.

Dessa maneira confirmando o estudo de Murray et al.^(25)^, o estudo-piloto realizado com duas crianças com transtornos dos sons da fala, sendo uma com atraso motor de fala e outra com apraxia de fala na infância, as quais passaram por intervenção intensiva do método ReST por doze sessões, demonstrou resultados satisfatórios ao tratamento.

Um estudo envolvendo revisão sistemática acerca dos tratamentos específicos para AFI, também demonstrou que ReST é clinicamente eficaz no tratamento de apraxia de fala na infância, pois fortes efeitos de generalização foram demonstrados no tratamento, evidenciando maior continuação dos efeitos do tratamento e generalização de pseudopalavras não tratadas^(26)^. A terapia com ReST através da aplicação do manual de intervenção também mostrou-se eficaz na modalidade de teleatendimento corroborando com estudos de Thomas et al.^(17)^ com cinco crianças de 5 a 11 anos com diagnóstico de AFI, as quais foram submetidas a intervenção terapêutica ReST por meio de videoconferência por doze sessões (quatro vezes na semana por três semanas). Os resultados mostraram aquisição significativa da imitação de pseudo-palavras selecionadas e generalização do efeito do tratamento para pseudopalavras não tratadas e palavras reais.

Sendo assim, após a finalização de todas as etapas, afirma-se que a tradução e adaptação do manual original em inglês para a língua portuguesa foi satisfatória, e se encontra adaptado para o contexto terapêutico clínico do Brasil, não sendo mais uma barreira linguística. Apesar das adaptações linguísticas terem sido contempladas, cabe ao clínico fazer adaptações fonéticas de acordo com a produção dos falantes da região, quando necessário. E com potencial para se tornar um recurso terapêutico de fácil acesso à prática clínica de fonoaudiólogos brasileiros, assim como no inglês e italiano.

Ressalta-se a importância de estudos de validação com uma amostra maior de crianças com design randomizados com os diferentes quadros clínicos, especificamente a apraxia de fala na infância e os transtornos motores de fala para verificar a eficácia do método.

## CONCLUSÃO

A partir dos estágios de tradução e com a aplicação do estudo piloto, foi possível verificar que o objetivo de traduzir e adaptar um programa de intervenção para crianças brasileiras com transtornos motores de fala foi atendido. Através da etapa de adaptação, foi verificada a necessidade da modificação de termos e instruções traduzidos para o português, pelos tradutores, para o contexto da prática clínica fonoaudiológica. O estudo-piloto também indicou necessidade de modificação no manual (Estágio 5), sendo necessário acrescentar mais um padrão de acentuação lexical, visto que no PB existem três padrões de acentuação lexical, e não dois como previsto no manual original em inglês. Quanto às organizações das sessões previstas no manual original não se tornou necessária modificações devido a boa aceitação dos pacientes. Dessa maneira o manual *Rapid Syllable Transition Treatment* (Rest) está adaptado para o contexto linguístico do português brasileiro, cabendo ao clínico adaptar, quando necessário, contemplando a variação linguística da região do paciente. Dessa maneira sugere-se novos estudos para comprovar a eficácia com uma maior amostra de crianças tanto do grupo clínico de transtorno motor de fala, quanto das demais alterações envolvendo os sons na fala.

## Appendix

Este artigo acompanha material suplementar.

Este material está disponível na página: https://rest.paginas.ufsc.br/
